# Unveiling the Antioxidant Profiling, Cytotoxicity and Wound Healing Potential of Biocompatible Silver Nanoparticles Synthesized Using *Solanum melongena*

**DOI:** 10.1002/fsn3.71569

**Published:** 2026-02-19

**Authors:** Dure Shahwar, Kinza Zafar, Mazhar Abbas, Fozia Anjum, Waqas Haider, Maha Gul Zafar, Muhammad Haseeb Zafar, Tariq Hussain, Ghulam Rasool, Muhammad Riaz, Hasan Ejaz, Quzi Sharmin Akter

**Affiliations:** ^1^ Department of Basic Sciences (Section Biochemistry) University of Veterinary and Animal Sciences (Jhang‐Campus) Jhang Pakistan; ^2^ Department of Gynecology and Obstetrics, Railways Hospital Islamic International Medical College Rawalpindi Pakistan; ^3^ Department of Chemistry Government College University Faisalabad Faisalabad Pakistan; ^4^ Medical Unit 3 Lahore General Hospital Lahore Pakistan; ^5^ Department of Basic Sciences (Section Pharmacology and Toxicology) University of Veterinary and Animal Sciences (Jhang‐Campus) Jhang Pakistan; ^6^ Department of Allied Health Sciences University of Sargodha Sargodha Pakistan; ^7^ Department of Clinical Laboratory Sciences, College of Applied Medical Sciences Jouf University Sakaka Saudi Arabia; ^8^ Department of Genetics and Animal Breeding, Faculty of Animal Science and Veterinary Medicine Patuakhali Science and Technology University Patuakhali Bangladesh

**Keywords:** fibroblast, free radicals, herbal remediations, wound repair

## Abstract

Medicinal plants play a vital role in wound healing and offer a viable solution to pathogen resistance to pharmaceuticals. Current study aimed to unveil the nutritional profile, antioxidant, cytotoxicity and wound healing potential of aqueous extract and synthesized silver nanoparticles of *Solanum melongena* . The crude extract was evaluated for nutritional profile via proximate analysis and determined the total flavonoids content (TFC) and total phenolic contents (TPC). The results showed higher nutritional value with 23.70 and 13.35 g/100 g DW of total fiber and crude protein content, respectively. The TPC and TFC analyses revealed the presence of significant phenolic and flavonoid content with 78.26 mg GAE/g DW & 89.93 mg CE/g DW at 90 mg/mL. Antioxidant potential was evaluated through DPPH assay, resulting in 68.67% (aqueous) and 81.87% (AgNPs) inhibition of free radicals. The promising antibacterial activity was shown by AgNPs against both Gram‐positive ( *B. subtilis* ) and Gram‐negative (*E. coli, P*. *vulgaris* , *S. typhimurium,* and *P. multocida)* bacterial strains, with a 19.5 mm ZOI recorded against *B. subtilis.* The biocompatibility was established through hemolytic assay exhibiting less than 5% hemolysis. The synthesized AgNPs of *S. melongena* were characterized, and SEM revealed a diameter of AgNPs as 30–52 nm. Through XRD analysis, average crystalline size was recorded as 15.37 nm and FTIR identified the key functional groups. LCMS analysis revealed the presence of key phytochemicals: campestral, cycloeucalenone, and neochlorogenic acid. The wound healing potential using crude and AgNPs was evaluated using eight groups of rabbits. Both extracts significantly reduced the wound size in rabbits, with nanoparticles showing higher efficacy. Histopathological studies revealed the reduced inflammation and markedly increased angiogenesis, fibroblast proliferation, re‐epithelialization and collagen deposition, confirming their potent wound‐healing activity. This study concludes that silver nanoparticles act as a potential carrier for drug delivery in targeted wounds, resulting in a significant reduction in wound size.

## Graphical abstract

This comparative study demonstrates the wound‐healing potential of aqueous and AgNPs‐synthesized *Solanum melongena* extracts. The eco‐friendly approach of AgNPs synthesis using plant extracts exhibit enhanced biocompatibility due to natural reducing agents that minimize toxicity and ensures safer and more effective biomedical applications. Phytochemical analysis confirmed substantial TPC and TFC, and LC–MS identified the key phytocompounds campestral, cycloeucalenone, and neochlorogenic acid. In vivo wound healing study showed that AgNPs result in higher wound‐size reduction than crude extract, accompanied by reduced inflammation and enhanced fibroblast proliferation, angiogenesis, collagen deposition, and re‐epithelialization.

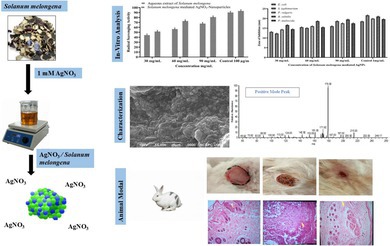

## 1 Introduction

Medicinal plants are widely used for burns and wounds, promoting natural healing by debridement, disinfection, and creating a moist environment to stimulate this process (Sharma et al. 2021). Plant‐derived bioactive compounds are gaining prominence, attributed to their potential antioxidant and antibacterial activities, serving as a natural alternative amid rising microbial drug resistance (Dadgostar 2019).

Skin injury, whether acute or chronic, initiates the body's healing processes, resulting in restoring its barrier function. Higher oxygen concentrations in open wounds increase ROS production, which leads to oxidative stress and impairs optimum wound healing (Tu et al. 2022). Antioxidant therapy using plants as free radical scavengers significantly promotes the healing process and accelerates various stages of it (Comino‐Sanz et al. 2021). The secondary metabolites contribute to its antioxidant potential and are beneficial in the oxidative stress‐related malignancies, including cancer, diabetes and CVD (cardiovascular disease). Conventional wound healing exists, but it faces limitations of inefficient drug delivery and efficacy and severe side effects (Tiwari and Pathak 2023). Topical antibiotics in the form of ointment are beneficial in bacterial infections; however, their application after the inflammation phase leads to hypersensitivity and allergic reactions. Its limited bioavailability restricts its application (Kolimi et al. 2022).

The green synthesis technique employing microorganisms and phytochemicals for the synthesis of nanoparticles (NPs) has emerged as a sustainable alternative (Tasic et al. 2022). Unlike the conventional synthetic methods, it offers plenty of advantages, including cost‐effectiveness, environmental friendliness, and no reliance on harmful chemicals, high temperatures, or pressing conditions. The synthesis of NPs has gained interest due to green synthesis owing to the negative effects of chemical and physical techniques (Duan et al. 2015). Furthermore, the application of nanoparticle‐based formulations for wound healing and infection control has increased remarkably (Nqakala et al. 2021), with their integration of nanoparticles into wound‐healing scaffolds recently garnering significant attention as a targeted and sophisticated dressing methodology (Negut et al. 2020). In wound‐healing scaffolds, the antimicrobial nanomaterials exist in diverse forms (liposomes, cellulose, and mesoporous silica), acting as antimicrobials and wound‐healing agents (Ren et al. 2022). Among antibacterial nanomaterials, metal‐ and metalloid‐based nanoparticles (MNPs) have demonstrated viable wound‐healing potential (Nethi et al. 2019). While numerous studies have assessed the antibacterial properties of various nanomaterials, only a few have focused on their specific application in enhancing the wound healing process.

Silver nanoparticles (AgNPs) possess distinctive physicochemical characteristics that make them valuable in biomedical applications, particularly wound healing and targeted drug delivery (Naganthran et al. 2022). Conventional wound healing exists, but it faces limitations of inefficient drug delivery and efficacy and severe side effects (Tiwari and Pathak 2023). Despite the promising potential of nanoparticles in disease and treatment, a key challenge hindering their clinical application is their long‐term persistence and potential toxicity (Kumar et al. 2024). The silver nanoparticles (AgNPs) used for biomedical or drug delivery purposes interact with blood cells and plasma proteins in the circulatory system; they may trigger undesirable pathophysiological responses (Yusuf et al. 2023). Hence, it is vital to evaluate the biocompatibility of these nanomaterials at the bio interface before clinical implementation (Kaiser et al. 2021). Exposure to cytotoxic particles results in cell membrane disruption and mitochondrial dysfunction (Wypij et al. 2021). While in the case of in vivo administration of NPs, these particles are rapidly adsorbed by competing serum proteins resulting in the formation of a protein corona (Das et al. 2021). Advanced studies on AgNPs revealed these nanoparticles can cause abnormal cardiac activity in zebrafish. The biocompatibility is strongly influenced by nanoparticle characteristics, including size, shape, surface functionalization, dosage, and concentration (Bullock and Bussy 2019). To mitigate these adverse effects and improve functionality, various coatings, including plant extracts, polymers, and proteins, are being used (Sathiyaseelan et al. 2020).

Vegetable by‐products obtained from the agro‐food industry, including peel, stem, leaf, seed, and kernel, are rich sources of fiber, minerals, vitamins, phytochemicals, and antioxidants (Hussain et al. 2023). Among these vegetables, *Solanum melongena* , or “aubergine,” is one of the popular species that belong to the same family, Solanaceae. *Solanum melongena* has a low‐fat content profile but is enriched with fibers, proteins, minerals, carbohydrates, vitamins, and phenolic compounds. The qualitative analysis of *S. melongena* fruit revealed the presence of phenols, terpenoids, alkaloids, flavonoids, steroids, saponins, and anthraquinones (Kellab et al. 2024). It is ranked as a top 10 vegetable in terms of antioxidant capacity and has attracted significant attention from consumers and experts throughout the globe due to its health advantages and is widely utilized for medicinal purposes (Kalloo and Bergh 2012).

Skin injury, whether acute or chronic, initiates the body's healing processes, resulting in restoring its barrier function. Higher oxygen concentrations in open wounds increase ROS production, which leads to oxidative stress and impairs optimum wound healing (Tu et al. 2022). Antioxidant therapy using plants as free radical scavengers significantly promotes the healing process and accelerates various stages of it (Comino‐Sanz et al. 2021). The secondary metabolites contribute to its antioxidant potential and are beneficial in oxidative stress‐related malignancies, including cancer, diabetes, and CVD (cardiovascular disease).

*S. melongena* leaves are used to clean wounds and as an astringent for bleeding disorders such as bladder hemorrhage (Anibarro‐Ortega et al. 2025). *S. melongena* L peels are by‐products in comparison to pulp and whole fruit and have high total phenolic compounds (Djouadi et al. 2016). The biological potential of *S. melongena* is attributed to phenolic compounds such as quercetin, chlorogenic acid, myricetin, p‐coumaric acid, cinnamic acid, and catechins (Kainat et al. 2023). Its phenylpropanoid pathway plays a key role in the production of secondary metabolites such as lignin and phenolic content (Talukder et al. 2025). Anthocyanins, an important group of natural phenolic compounds (Djouadi et al. 2016), including nasunin (delphinidin‐3‐p‐coumaroylrutinoside‐5‐glucoside), a major compound (Gallo et al. 2014), possess the most potent antioxidant activity as determined by in vitro analysis. Other anthocyanins include delphinidin‐3‐glucoside, delphinidin‐3‐rutinoside, petunidin‐3‐rutinoside, and cyanidin‐3‐rutinoside. Chlorogenic acid is the main phenolic substance present in all *Solanum melongena* types (Nayanathara et al. 2016). Its extraction with purple skin is very effective in scavenging superoxide radicals and inhibiting the hydroxyl radicals production through the chelation of ferrous iron (Sharma et al. 2019). Its extracts have been reported to successfully suppress the development and growth of tumors and lung cancer (Sharma et al. 2025), inhibit inflammation (Kellab et al. 2024), and cardiovascular diseases (Hazra 2023).

Based on its antioxidant and antibacterial activity, our current research work aimed to comparatively investigate the potential of *Solanum melongena* aqueous and green synthesized silver nanoparticles (AgNPs) for wound healing through in vitro and in vivo analysis. The extracts were analytically characterized by FTIR, XRD, and scanning electron microscopy (SEM). The proximate analysis estimates the nutritional contents to assess the quality, safety, and nutrient content and ensure food quality and balanced animal nutrition. The extract was evaluated for its antioxidant potential and antimicrobial properties. Key phytoconstituents were identified by LCMS analysis. Both extracts were evaluated for in vivo wound healing potential using rabbits as model animals.

## 2 Materials and Methods

### 2.1 Chemicals and Reagents

All the reagents and chemicals were of analytical grade used during the whole experimental work. All these, methanol, silver nitrate (AgNO_3_), 2,2‐diphenyl picrylhydrazyl reagent (DPPH), Folin–Ciocalteu (FC) reagent, sodium carbonate (NaCO_3_), gallic acid (GA), aluminum chloride (AlCl_3_), sodium hydroxide (NaOH), quercetin, and ascorbic acid (AA), xylocaine, and polyfax were purchased from commercial companies, Sigma Aldrich and Merck Germany.

### 2.2 Collection and Extraction

The *Solanum melongena* (eggplant) fruit was collected from AARI (Ayub Agriculture Research Institute, Faisalabad, Pakistan). Peels were removed from eggplant using a sharp knife and disinfected using 2.5% solution of sodium hypochlorite for 5 min and then rinsed with water. About 3 g of peel was homogenized in 30 mL of water then left for 1 h under constant stirring at 35°C in an orbital shaker. Subsequently, simple filtration was performed, recovering the extract and stored at room temperature in the dark until use (Estrella‐Osuna et al. 2022).

### 2.3 Biosynthesis of AgNPs

Nanoparticle synthesis was carried out following the protocol described by Vanti et al. (2020) with some modifications. The aqueous extract was mixed with an equal ratio (1:1) of 1 mM solution of silver nitrate, followed by continuous stirring. The complete experimental setup was incubated for 12 h at room temperature under continuous stirring at 240 rpm. Change in color from milky white to brown indicates nanoparticle synthesis and then confirmed spectrophotometrically. Nanoparticles were collected by centrifugation and air dried for further bioassays.

### 2.4 Characterization of Synthesized Silver‐Nanoparticles

The FTIR spectra of *Solanum melongena* extract were measured using a German‐made FTIR instrument (Model/Make: IFS25, Bruker), operated by PC‐based software. The information on infrared transmittance was gathered using wave numbers between 4000 and 500 cm^−1^ (Fadlelmoula et al. 2022). The Jeol JSM‐6480 LV SEM was utilized to assess the mean particle size and shape of *Solanum melongena* AgNPs. The vacuum used to run the Jeol JSM‐6480 LV SEM machine was in the order of 10^−5^ Torr, and the microscope's accelerating voltage was maintained between 10 and 20 kV (Koga et al. 2021). *S. melongena* AgNPs were examined using an X‐ray Diffraction Unit (XRD) (Pan Analytical, X‐pert Pro, and Netherlands). X‐ray diffraction (XRD) of *S. melongena* AgNPs was measured using a Cu‐K radiation source with a scattering range of 20–80 θ on an apparatus running at 45 kV and 40 mA. The technique was used to determine the existence of crystalline make‐up, phase variety, and grain size of the produced silver nanoparticles. Using Scherrer's equation, the prepared samples' particle size was calculated (Jemal et al. 2017).

$$\text{Crystallinity}=\frac{\text{Area of crystalline peak}}{\text{Area of}\;\mathrm{all}\;\text{peaks}\;\left(\text{crystalline and amorphous}\right)}$$

D=kλ/βcosθ

In this context, *K* denotes the shape factor, β represents the full width at half maximum (FWHM) of different peaks, and λ is the wavelength of the X‐rays, whereas θ corresponds to Bragg's angle.

### 2.5 Identification of Key Phytoconstituents by LCMS Analysis

Liquid chromatography coupled with mass spectrometry was used to identify the phytoconstituents present in the *Solanum melongena* L extract. First, a multichannel solid phase extraction (SPE) cartridge with Strata C18 columns and a vacuum pump was used to prepare the sample. For LCMS analysis, an Agilent 1290 Infinity LC system coupled with a Q‐TOF 6520 mass spectrometer using an ESI source was employed for positive and negative mode ionization. Chromatic separation was performed utilizing a Zorbax Eclipse XDB‐C18 column at 25°C using two mobile phases: A‐0.3% formic acid and B‐90% of acetonitrile in natural solvent. During LCMS analysis, the RT value (retention time) of phytoconstituents, corresponding with the mass‐to‐charge ratio in the mass spectrum and relative intensity, was recorded. MS signals were detected throughout the chromatographic separation process, and comprehensive results were recorded (Rahim et al. 2022).

### 2.6 Proximate Analysis

Proximate analysis of *Solanum melongena* plant was performed following the method described by AOAC (Choudhury et al. 2020). Moisture content was determined by the oven drying method (Carneiro et al. 2018). About 4 g of *Solanum melongena* fruit powder was weighed and placed in a hot air oven at 90°C for 12 h. After cooling, the sample was weighed again. Protein concentration in the fruit extract of *Solanum melongena* was determined by the Kjeldhal method. For determination of ash contents in a muffle furnace, a silica crucible was heated and cooled in a desiccator. After weighing the initial weight, 5 g of sample was heated in a muffle furnace at 550°C for 6 h (Sultana 2020). Soxhlet extraction apparatus was used to determine the crude fat. Then 2 g of *Solanum melongena* sample was weighed and placed in Soxhlet apparatus for 2 h, which has n‐hexane solvent. The sample was put in the oven for 10 min at 90°C. After 10 min, the dried sample was weighed and the value of crude fat calculated (Hewavitharana et al. 2020). Crude fiber in the fat‐free fruit extract of *Solanum melongena* was determined by the digestion method (Zhou et al. 2018).

### 2.7 Preparation of Stock Solution

The stock solution of three concentrations, 30, 60, and 90 mg/mL, of crude and nano‐synthesized extracts of *S. melongena* peel was prepared to carry out antioxidant analysis, including TFC, TPC, and DPPH radical scavenging activity.

#### 2.7.1 Antioxidant Analysis

The quantitative analysis of the aqueous extract carried out for the determination of phenolic and flavonoid contents. The radical scavenging potential was carried out by DPPH assay. In these analyses, extracts at three different concentrations, that is, 30, 60 and 90 mg/mL, were used. Phenolic contents of *Solanum melongena* aqueous extract was measured using Folin–Ciocalteu reagent according to the spectrophotometric method and flavonoid contents using the AlCl_3_ colorimetric method as described by Abbas et al. (2025). The results were expressed as mg of catechin equivalent per g dry weight (mg CE/g DW) for TFC and mg of gallic acid equivalent per g dry weight (mg GAE/g DW) for TPC. The free radical scavenging potential of aqueous extract and synthesized AgNPs was carried out using DPPH assay, and for that purpose, a 0.004% methanolic DPPH solution was prepared. About 5 mL of prepared DPPH solution was mixed with 50 μL of each concentration of samples and incubated for 30 min in the dark at room temperature. The absorbance was then taken at 517 nm by using ascorbic acid as standard, as described earlier (Zafar et al. 2025). Ascorbic acid and quercetin (100 μg/mL) both served as control.

$$\text{DPPH};\%\text{of antioxidant activity}=\left[\left(\mathrm{Ac}-\mathrm{As}\right)÷\mathrm{Ac}\right]\times 100$$

where Ac is the control absorbance and As is the sample absorbance.

#### 2.7.2 Antioxidant Enzyme Analysis

The antioxidant potential of both aqueous extract and silver nanoparticles of *Solanum melongena* was evaluated through in vitro antioxidant enzyme assays. Enzyme extracts were prepared by homogenizing the extracts in appropriate buffer solutions followed by centrifugation for 20 min at 15,000 rpm. The resulting supernatant served as the enzyme source, prepared at various concentrations (30, 60, and 90 mg/mL). The Catalase (CAT) activity of both extracts was evaluated using standard protocol (Dulta et al. 2022). The reaction mixture was prepared comprising 50 μL of 0.3% H_2_O_2_ with 0.1 mL of enzyme extract, and the final volume was adjusted to 3 mL with 50 mM phosphate buffer. The reduction in absorbance at 240 nm was recorded at 15‐s time intervals for 5 min using a UV–vis spectrophotometer. Superoxide dismutase (SOD) activity was assessed by following the method of Naz et al. (2022). For this assay, the reaction mixture was prepared by mixing 0.3 mL of enzyme extract, 2.75 mL of Tris–HCl buffer, and 60 μL of pyrogallol. The mixture was incubated for 10 min at 25°C. Then, a 500 μL aliquot was transferred to a cuvette, and absorbance was measured at 420 nm using a UV–vis spectrophotometer. The peroxidase (POD) activity was determined by following the method of Aftab et al. (2024). Briefly, 0.3 mL of enzyme extract was mixed with 2.4 mL of phosphate buffer, followed by the addition of 0.6 mL of guaiacol. Subsequently, 20 μL of 5% hydrogen peroxide (H_2_O_2_) was added, and the mixture was incubated at room temperature for 30 min. Enzyme activities were expressed as IU/mL of protein.

#### 2.7.3 Antibacterial Activity

Antibacterial activity of crude and synthesized silver nanoparticles of *S. melongena* was analyzed against *Escherichia coli, Proteus vulgaris, Salmonella typhimurium, Bacillus subtilis* and *Pasteurella multocida* (Urnukhsaikhan et al. 2021). Nutrient agar was freshly prepared, and 30 mL of agar was poured into already labeled petri plates. About 200 μL of bacterial culture of 1.2 × 10^8^ CFU/mL of cell density was evenly spread over the agar surface, followed by 30 min incubation. After the solidification of agar media, wells were induced using a 6 mm sterile cork borer. Afterwards, 50 μL of plant extract and nanoparticles of varying concentrations (30, 60 and 90 mg/mL) were poured while Ciprofloxacin (1 mg/mL) was added as a control. Petri plates were incubated at 37°C for 24 h, and inhibition zones were measured using a zone reader.

### 2.8 Cytotoxicity Analysis by Hemolytic Assay

The safety profile of crude and synthesized silver nanoparticles of *S. melongena* was assessed using a hemolytic assay by following the method of Abbas et al. (2018) with slight modification. The blood was collected in heparinized evacuated tubes, washed thoroughly with phosphate buffer saline (PBS, pH 7.4), and centrifuged at 4000 rpm for 5 min. Washing with PBS was repeated 3–5 times to remove non‐erythrocytic components. The resultant RBCs were suspended in the chilled PBS (20 mL). Using a microscope, RBCs were counted and adjusted at a standardized concentration of 7.068 × 10^8^ cells/mL. Then, 180 μL of RBC suspension was mixed with 20 μL of test solution (30–90 mg/mL). The reaction mixture was incubated at 37°C for 30 min with constant agitation. The samples were gently mixed and centrifuged at 1310 g for 5 min. Then, 100 μL of the supernatant was taken, diluted with 900 μL of PBS, and the absorbance was measured at 576 nm using a UV–Vis spectrophotometer (Shimadzu UV‐1800). The PBS (negative control) and 0.1% Triton X‐100 (positive control) were used.

$$\%\text{Lysis of erythrocyte}=\left(A_{\text{sample}}/A_{\text{triton}\;X-100}\right)\times 100$$

### 2.9 Wound Induction and Healing Potential

The in vivo wound healing model involved the use of healthy male rabbits. The Ethical Committee, College of Veterinary and Animal Sciences (CVAS), Pakistan, gave its prior approval. For the investigation, 24 healthy male rabbits with average weights of 2–2.5 kg were chosen and divided into eight groups, 3 in each. Animals were maintained for 24–48 h from 12 h photoperiod, 25°C, access to food and tap water under observation. Under the supervision of the Surgery Section of the Department of Clinical Sciences, CVAS, Jhang, wounds were induced. Each rabbit's fur at the dorsal surface of the lumbar region was shaved prior to wound induction. Open skin incisions measuring three centimeters were made on the dorsal surface of the lumbar region using a standard aseptic technique. The dorsal side of the lumbar region (3 × 3 cm) is sliced using sterile scalpel blades to open the incision. Animals were kept under observation. The anesthesia xylocaine (3 mg/Kg b.w) was administered subcutaneously. After a latency period of about 3–5 min, wound induction was performed (Abbas et al. 2022). Using the below equation, the wound's size was calculated.

$$\text{Wound reduction}\%=\frac{\text{Wound area}\;\mathrm{Day}\;0-\text{Wound area}\;\mathrm{Day}\;T}{\text{Wound area}\;\mathrm{Day}\;T}\times 100$$

The study animals were divided into following groups: Groups 1 and 2 were positive (Polyfax) and negative controls (untreated group), respectively. Group‐3, Group‐4, and Group‐5 receive 30, 60 and 90 mg/mL of *S. melongena* crude aqueous extract, respectively while Group‐6, Group‐7, and Group‐8 receive the same concentrations of *S. melongena* mediated silver nanoparticles, respectively.

### 2.10 Histopathological Study

Histological studies of tissues were carried out to track the healing process. Tissues from the healing wounds were removed and preserved in neutrally agitated formalin for a period of 3 days. The samples were processed in different concentrations of xylene and alcohol after being washed. Every tissue sample was placed into a block for storage after being embedded in paraffin wax. A microtome was used to cut each block to a thickness of 0.5 mm. The slides were prepared and examined under a microscope using Hematoxylin and Eosin dyes (Suvaneeth et al. 2018).

### 2.11 Statistical Analysis

All experiments were performed in triplicate, and the results were presented as mean ± standard deviation. Antioxidant and antimicrobial data were statistically evaluated by applying analysis of variance using GraphPad Prism. A significance threshold of *p* ≤ 0.05 was set to assess statistical significance, and Tukey's post hoc test was employed to determine specific pairwise differences among the treatment groups for wound healing (Jangid et al. 2024).

## 3 Results and Discussion

### 3.1 Characterization of Synthesized Silver‐Nanoparticles

#### 3.1.1 SEM Analysis

**FIGURE 1:**
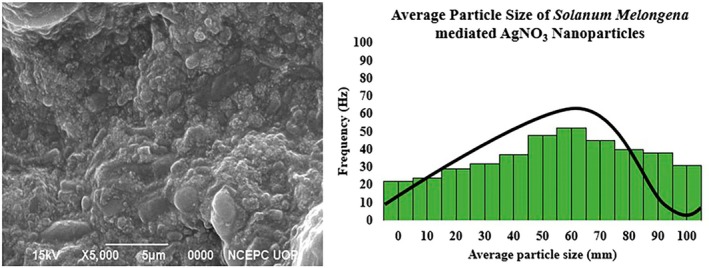
Morphological (SEM) images of *S. melongena* mediated AgNPs.

Scanning electron microscopy measurements were used to determine the morphology and size details of synthesized silver nanoparticles. The representative SEM image was recorded at a magnification of 5000× with 15 kV accelerating voltage for synthesized AgNPs. The SEM image (Figure 1) gives the appearance of an overall uniform surface of nanoparticles. However, the individual particles are not uniform but rather exist in aggregated form, making large secondary structures. While the analysis of data through SEM micrographs shows the high density of silver nanoparticles in the diameter range of 30–52 nm along the particle size distribution histograms using Image J software. Under the observations of an optical microscope, it was observed that these spherical‐shaped green synthesized AgNPs were not in direct contact even after aggregation, indicating their stabilization by phytoconstituents as capping agents (Devi and Joshi 2015; Geoprincy et al. 2013). Silver nanoparticles synthesized using *Acalypha indica* leaf extracts have been characterized by Krishnaraj et al. (2010) and also observed the analogous findings.

#### 3.1.2 XRD Analysis

**FIGURE 2:**
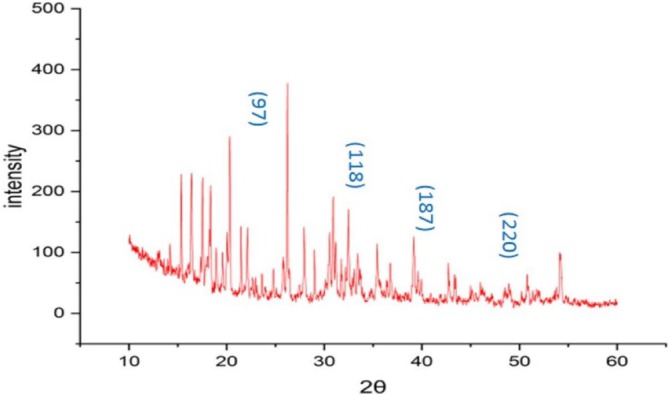
XRD diffractogram of synthesized *S. melongena* mediated AgNPs.

For phase determination of the crystalline structure of nanoparticles, the AgNPs were subjected to powder XRD (Table 1). Figure 2 shows the XRD patterns of synthesized AgNPs using *S. melongena* peel extract. Four intense peaks were indexed at peak positions (2θ) of 38.53°, 44.32°, 52.48°, and 60.08° with lattice planes (hkl) of (1 2 2), (1 1 1), (2 0 0), and (2 2 0), respectively. Table 1 gives the average crystalline sizes and lattice planes of four peaks. The average crystalline size of green synthesized AgNPs was calculated to be 15.37 nm using the Debye‐Scherer equation. The representation of sharp diffraction peaks was attributed to the crystalline nature of synthesized AgNPs due to the thick organic coating of phytoconstituents (Logeswari et al. 2015; Shaik et al. 2018). A similar diffraction pattern is also observed by Pushparaj et al. on XRD analysis of AgNPs synthesized using *S. melongena* leaves and concluded that all the diffraction peaks correspond to the characteristic face‐centered cubic (FCC) silver lines and spherical shape of nanoparticles (Pushparaj et al. 2023).

**TABLE 1:** XRD results of synthesized AgNPs using *S. melongena* peel extract.

Peak position (2θ)	Theta (θ)	FWHM (β)	Crystallite size D (nm)	d‐spacing (Å)	Lattice planes (hkl)
38.53	19.265	0.76	11.07	2.33	(1 2 2)
44.32	22.16	0.53	16.18	2.04	(1 1 1)
52.48	26.24	0.44	20.12	1.74	(2 0 0)
60.08	30.04	0.65	14.11	1.53	(2 2 0)

*Note:* Average crystalline size D (nm) of green synthesized AgNPs = 15.37 nm.

#### 3.1.3 FTIR Analysis

**FIGURE 3:**
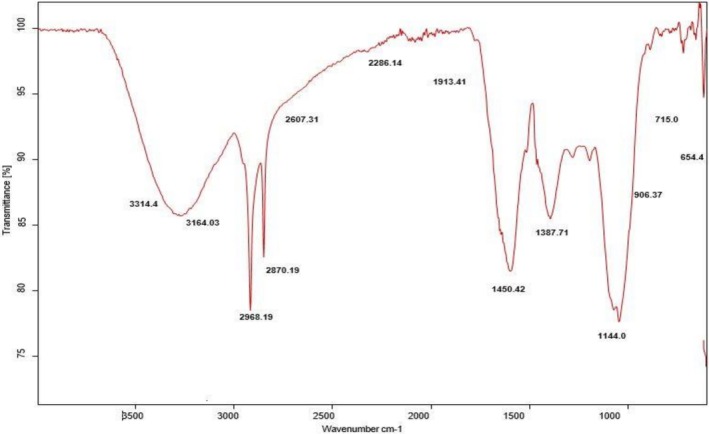
FTIR spectra showing functional groups in *S. melongena aqueous* extract.

FT‐IR spectroscopy was used for the evaluation of phytochemicals' surface functional groups in an aqueous extract of *S. melongena* peels. Figure 3 showed a broad absorption band in the high energy region 3500–3000 cm^−1^, characterizing the OH stretching of phenolic and flavonoid compounds (Selvan et al. 2018). By comparing with the FTIR spectrum of *S. melongena* observed previously by Das and Bhuyan (2019), the medium absorption peaks in the region of 2500–2000 cm^−1^ are only observed in the present analysis and were attributed to the C‐H stretching and bending vibrations of aromatic compounds in *S. melongena* peels. While a sharp absorption peak at 1636.36 cm^−1^ is attributed to the stretching of carbonyl groups in amide linkages, suggesting that proteins are also involved in the synthesis of silver nanoparticles. These observations revealed that the carbonyl group of amino acids in proteins can act as capping agents, which prevents the agglomeration of synthesized silver nanoparticles to provide them stability (Boopathi et al. 2012). The weak absorption band at 990 cm^−1^ corresponds to the bending vibrations of the C=C bond of terpenes (Amponsah et al. 2022). The short intense bands observed in the region 726–611 cm^−1^ show the presence of heterocyclic compounds due to the occurrence of flavonoids. The sharp peak at 613.69 cm^−1^ in the finger print region was attributed to the occurrence of low molecular weight carbohydrates (Cao et al. 2017). The overall observation proves the involvement of a variety of phytochemicals, such as anthocyanin, delphinidine‐3‐(p‐cumaroylrutinoside)‐5‐glucoside, and so on, as reducing agents, and proteins may act as stabilizing agents (Sultana and Misbahuddin 2020). The above observations were in line with (Sharma et al. 2016), who carried out the biological synthesis of silver nanoparticles using *S. melongena peel* waste as a green source and found that the phytoconstituents are used as capping agents to stabilize the nanoparticles via FTIR analysis.

#### 3.1.4 LC–MS/MS Analysis

**FIGURE 4:**
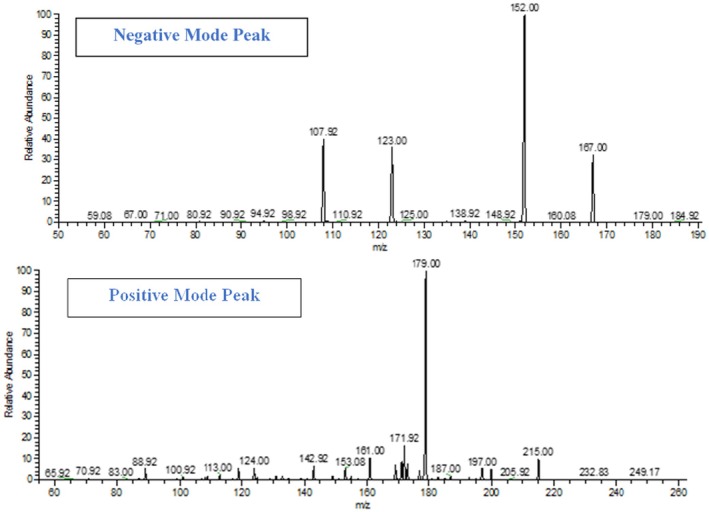
Peaks from positive and negative mode analysis of LCMS/MS.

By comparing the fragmentation patterns (i.e., product ions) obtained by LCMS analysis with the molecular (precursor) ion, various bioactive compounds were identified in *Solanum melongena L* given in Table 2 and shown in Figure 4. The reported literature databases (PDB), and computer repositories (NIST and European mass data bank) for reference compounds were then compared with the LC–MS results. Peak 8 (RT 0.49) shows a peak at m/z 401.7, indicating the presence of campestral, which belongs to the phytosterol class. Peak 1 (RT 0.86) shows a peak at m/z 425.9, indicating the presence of cycloeucalenone. Peak 2 (RT 1.14) shows a peak at m/z 355.3, demonstrating the presence of neochlorogenic acid. Peak 4 (RT 1.24) at m/z 300 represent the N‐trans‐p‐coumaroyl octopamine. Peak 7 (RT 4.63) at m/z 453.4, indicate the presence of Torvanol A. Peak 19 (RT 4.04) at m/z 580.5 indicate the presence of Pelargonidin 3‐rutinoside. Peak 13 (RT 3.87) at m/z 181.1 demonstrate the presence of caffeic acid. Peak 5 (RT 2.87) at m/z 303.43 indicate the presence of quercetin. Peak 12 (RT 2.76) shows a peak at m/z 355.31, indicating the presence of chlorogenic acid. The phytochemicals of *Solanum melongena L*. belong to five major classes, namely free reducing sugars, phenolic acids, anthocyanins, glycoalkaloids, and amide proteins. In this chromatograph, peaks 12, 2, and 6 belong to the phenolic acid class, namely chlorogenic acid, neochlorogenic acid, caffeic acid, and methyl caffeate, respectively. Peaks (19, 16, and 13) indicate the anthocyanin class, namely Pelargonidin 3‐rutinoside, Nasunin, and Petundin‐3‐rutinoside, respectively. Peak 14 indicate the presence of the flavonoid glycoside class, namely p‐coumaroylrutinoside. Peaks (1 and 5) indicate the presence of terpene classes, namely cycloeucalenone and torvanol A, respectively. Peak 4 indicates the presence of an alkaloid class, namely N‐trans‐p‐coumaroyl octopamine. These results provide insights into the concentrations and molecular structures of these compounds, supporting for the characterization and identification within the sample analyzed.

**TABLE 2:** LCMS/MS analysis of *Solanum melongena L* peel extract.

Compound name	m/z	Intensity	R.T value	Structure	Molecular mass	Biological role	References
Campestral	401.7	347 56 89	0.49	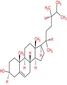	400.7	It has anti‐inflammatory activity, used for treatment of rheumatoid arthritis	Nazir et al. (2023)
Cycloeucalenone	425.9	56 88 143	0.86	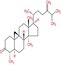	424.7	It is for treatment of fever, diabetics and hypertension.	Kongkathip et al. (2002)
Neochlorogenic acid	355.3	76 234 89	1.14	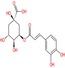	354.31	It has anti‐inflammatory and anti‐tumor role.	Che et al. (2021)
N‐trans‐pcoumaroyl octopamine	300.2	134 78 98	1.24	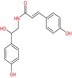	299.32	It is used for treatment of Alzheimer.	Roumani et al. (2020)
Torvanol A	453.4	45 145 342	4.63	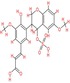	452.4	It has anti‐viral and anti‐ antidepressant.	Mohan et al. (2013)
Pelargonidin 3‐rutinoside	580.5	345 432 143	4.04	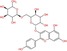	579.5	It has anti‐oxidant and anti‐cancer activity.	Mullen et al. (2002)
Caffeic acid	181.13	87 98 123	3.87	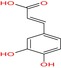	180.16	It has anti‐inflammatory, anti‐oxidant and anti‐cancerous potential.	Espíndola et al. (2019)
Methyl cafeate	195.14	78 98 123	3.46	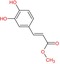	194.18	It has anti‐oxidant, neuroprotective and anti‐cancerous potential.	Jantas et al. (2020)
Nasunin	956.5	874 543 345	3.32	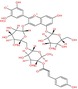	955.3	It has antioxidant activity prevent food allergies.	Gallo et al. (2014)
Delphinidin 3‐glucoside	466.4	56 98 342	3.12	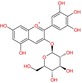	465.4	It suppresses breast cancer cells and cardioprotective	Yang et al. (2016)
p‐coumaroylrutinoside	956.4	765 543 233	2.98	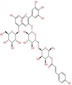	955.3	It is cytoprotective antioxidant.	Li et al. (2017)
Gallic acid	171.14	98 76 45	2.67	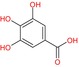	170.12	It is strong antioxidant and have cardioprotective potential.	Gao et al. (2019)
Cyanidin‐3‐rutinoside	596.7	134 453 76	2.45	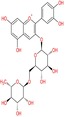	595.5	It has antioxidant, antihyperglycemic and cardioprotective potential	Thilavech and Adisakwattana (2019)
Petunidin‐3‐rutinoside	626.6	233 78 432	2.06	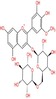	625.6	It has antioxidant and anti‐inflammatory activity.	Nemzer et al. (2020)
Chlorogenic acid	355.31	123 87 321	2.76	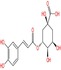	354.31	It has antioxidant, antibacterial, anti‐inflammatory, and antiviral potential.	Yu et al. (2022)
Quercetin	303.43	43 76 45	2.87	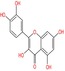	302.23	It has anti‐oxidant and cardioprotective potential	David et al. (2016)

### 3.2 Proximate Profiling

The proximate composition of the fresh peels of *S. melongena* is presented in Table 3. The results indicate a high proportion of moisture (44.25 ± 0.40 g/100^−g^ FW) as compared to that of peels of other fruits reported by Morais et al. (2017). The high moisture content is an indicator of being classified *S. melongena* among the most perishable fruit, but this property makes its peels and pulp beneficial to people suffering from constipation, carcinoma of the colon and rectum, diverticulitis, and atherosclerosis (Chinedu et al. 2011). However, the crude protein content was obtained to be 13.35 ± 0.56 Kcal/100/100^−g^ FW, which is in good agreement with the previous studies of Zia‐ur‐Rehman et al. (2003) who reported the values of 12.4% and 11.5%, respectively for protein content in the peels of *S. melongena* . Although the protein contents in *S. melongena* peels are low, they are still useful in the repair of worn‐out tissues in the body (Mohamed et al. 2019). The crude fat content of 12.56 ± 0.34 g/100^−g^ FW was obtained for fresh peels of *S. melongena* , which falls in line with the fat content evaluated from *S. melongena* peel reported by Hossain et al. (2015). For dietary fiber analysis, the highest total fiber contents (23.70 ± 0.51 g/100^−g^ FW) were evaluated, similar to fiber content obtained by Scorsatto et al. (2017) in fresh dried peels. Therefore, *S. melongena* peels may be used to produce functional or fiber fortified food. The ash content (6.14 ± 0.52 g/100^−g^ FW) was comparable to the contents observed in the peels of pineapple (4.39% ± 0.14%) and sweet orange (4.89% ± 0.06%) by Egbuonu and Osuji (2016) and Romelle et al. (2016), respectively.

**TABLE 3:** Nutrient concentration in *Solanum melongena* peel.

Composites	Concentration
Moisture	44.25 ± 0.40 (g/100^−g^ FW)
Crude Protein	13.35 ± 0.56 (Kcal/100^−g^ FW)
Crude Fat	12.56 ± 0.34 (g/100^−g^ FW)
Total Fiber	23.70 ± 0.51 (g/100^−g^ FW)
Ash	6.14 ± 0.52 (g/100^−g^ FW)

*Note:* Values are expressed as mean ± SD.

### 3.3 Antioxidant Activity

**FIGURE 5:**
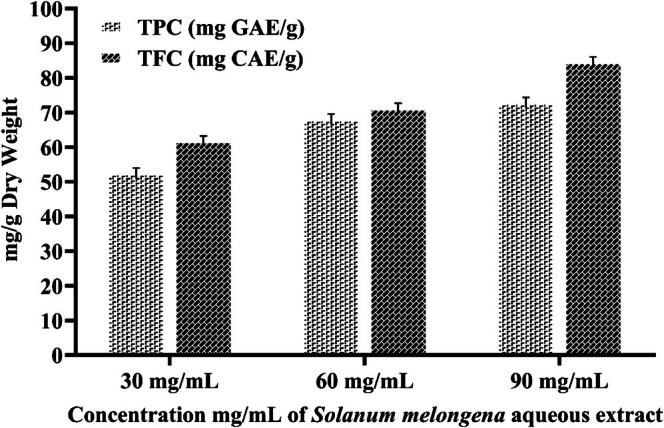
Total phenolic and flavonoid content analysis of *S. melongena* aqueous extract.

**FIGURE 6:**
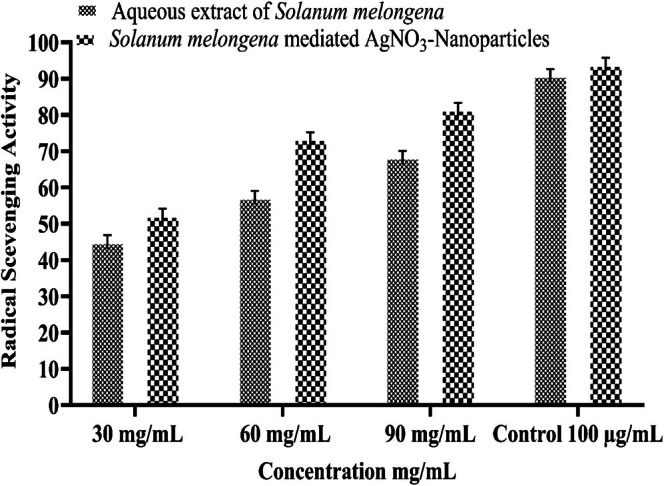
Radical scavenging potential of crude and green synthesized *S. melongena* extract.

The results of the phenolic and flavonoid content analysis of the *S. melongena* aqueous peel extract show a dose‐dependent trend as shown in Figures 5 and 6. At low concentration (30 mg/mL), extract exhibited 51.86 mg GAE/g DW phenolic content, which reaches 78.26 mg GAE/g DW at 90 mg/mL. A similar trend was observed in TFC, showing 59.1–89.93 mg CE/g DW flavonoid content over the concentration range of 30–90 mg/mL. These results were corroborated with the findings of Afreen et al. (2020), citing higher total phenolics and flavonoids in green synthesized AgNPs as compared to crude extract. Furthermore, AgNPs synthesized using *Clinacanthus nutans* exhibited 75.52 ± 0.905 μg GAE/mg and 27.97 ± 1.273 μg QE/mg as TPC and TFC, respectively (Mat Yusuf et al. 2020). Another study by Kona et al. (2024), reported the phenolics and flavonoids of green AgNPs as 250.14 μg GAE/g and 890.56 μg QE/g extract, respectively. While results from the DPPH assay exhibited a higher scavenging potential by increasing the concentration for both aqueous extract and synthesized AgNPs. It was observed that synthesized AgNPs showed increased antioxidant activity (% inhibition 73.76 ± 1.29 and 81.87 ± 1.21), followed by aqueous extract (57.59 ± 1.6 and 68.67 ± 1.17) at 60 and 90 mg/mL concentrations, respectively. While both the ascorbic acid and quercetin, at 100 μg/mL, showed 91.2% and 94.3% radical scavenging activity, respectively. The substantial levels of phenolics and flavonoids in *S. melongena* peel are attributed to higher percentage inhibition values of AgNPs. Ascorbic acid and quercetin used as positive control in 100 μg/mL concentration. These compounds function as capping or stabilizing agents to improve the scavenging capacity of biosynthesized AgNPs. Furthermore, a prior study that used a mixed extract of *Pandanus foetidus and Alangium salvifolium* peels to biosynthesize AgNPs showed better scavenging capacity than a simple extract of peels (Das et al. 2019). The above result strongly suggested that green‐synthesized AgNPs can be used as potential antioxidants.

#### 3.3.1 Antioxidant Enzyme Analysis

**FIGURE 7:**
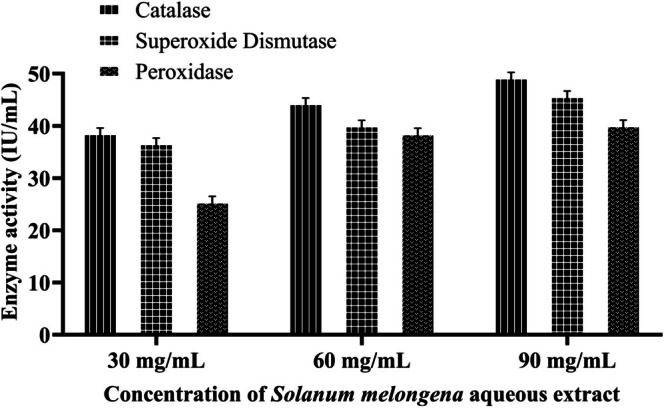
Antioxidant enzyme analysis of *S. melongena* aqueous extract.

**FIGURE 8:**
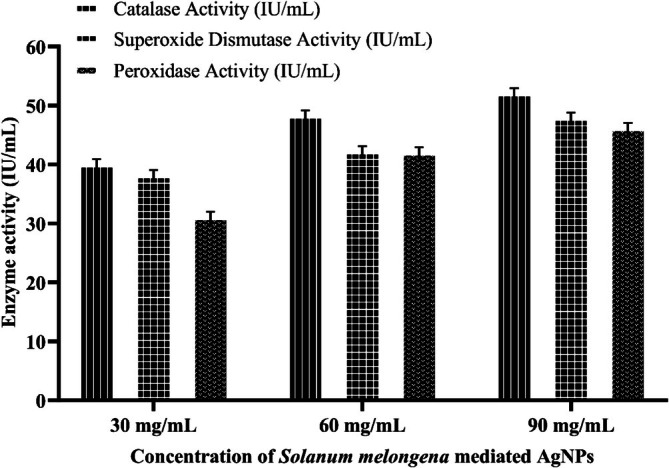
Antioxidant enzyme analysis of *S. melongena* mediated AgNPs extract.

At 30 mg/mL, catalase, superoxide dismutase, and peroxidase activities for aqueous extract were recorded as 38.83 ± 0.89, 36.93 ± 0.94, and 25.75 ± 0.79 IU/mL, respectively as shown in Figure 7. For AgNPs, it is exhibited at 40.13 ± 1.09, 38.28 ± 0.7, and 31.2 ± 0.95 IU/mL for catalase, superoxide dismutase, and peroxidase, respectively (Figure 8). The antioxidant enzyme content recorded for AgNPs is slightly higher than aqueous extract. These results increased to 44.58 ± 1.05, 40.31 ± 1.02, and 38.82 ± 0.82 IU/mL for aqueous extract with increasing concentration up to 60 mg/mL, indicating a significant improvement, especially in peroxidase activity. AgNPs showed 52.12 ± 1.21 IU/mL for catalase at the highest tested concentration of 90 mg/mL, compared to 49.49 ± 1.02 IU/mL for superoxide dismutase and 45.92 ± 1.05 IU/mL for aqueous extract. AgNPs are measured at 46.21 ± 0.91 IU/mL and peroxidase activity is reported as having increased to 40.37 ± 0.91 IU/mL. Plants' antioxidant defense mechanism protects against environmental stress and scavenges excess ROS, protecting plant cells from protein damage, lipid peroxidation, and DNA degradation. POD scavenges H_2_O_2_ in the vacuoles, CAT transforms hydrogen peroxide into water and oxygen, and SOD eliminates oxygen free radicals (Dumanović et al. 2021; Fujita and Hasanuzzaman 2022). These results reveal that *S. melongena* acts as a natural antioxidant against ROS‐induced oxidative stress. Our results corroborate (Riaz et al. 2021), who reported that leaves of *S. melongena* act as major natural antioxidants. Another study conducted using ZnO NPs of *Carica papaya* demonstrated enhanced superoxide dismutase and catalase activity attributed to ZnO NPs significantly impacting antioxidant metabolism during seed germination (Dulta et al. 2022).

### 3.4 Antibacterial Activity

**FIGURE 9:**
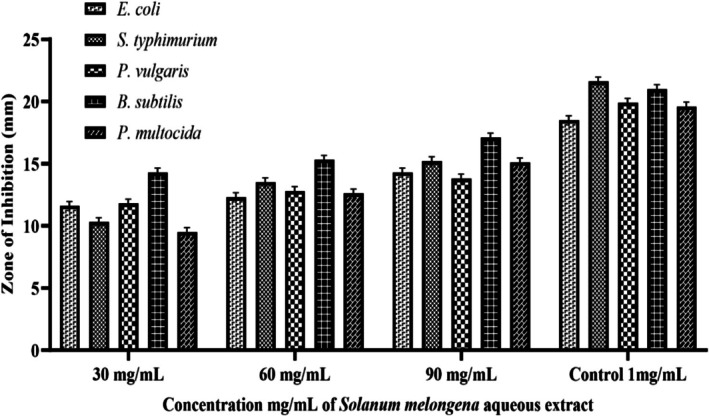
Antibacterial activity of *S. melongena* aqueous extract.

**FIGURE 10:**
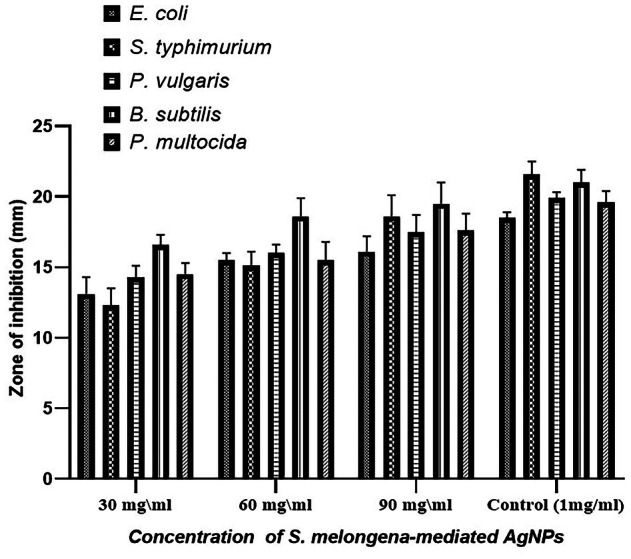
Antibacterial activity of *S. melongena* ‐mediated AgNPs.

The disk diffusion assay revealed that inhibition potential against selected strains was highly dependent on the concentration of aqueous extract and Ag_3_NPs (Figures 9 and 10). It was observed that synthesized AgNPs showed greater antibacterial potential based on greater inhibition zones tested for both Gram‐positive ( *B. subtilis* ) and Gram‐negative (*P. multocida, S. typhimurium, E. coli, and P. vulgaris*) strains using Ciprofloxacin as a standard antibiotic. With zone of inhibition (ZOI) values of 19.5, 18.6, and 17.6 mm (exhibited by synthesized AgNPs), the greatest concentration (90 mg/mL) demonstrated considerable bacterial inhibitory action against *B. subtilis, S. typhimurium, and P. multocida*. However, the aqueous extract from *S. melongena* peels showed 17.1, 15.2, and 15.5 mm. In contrast, the inhibitory zones of 17.5 and 16 mm and 13.8 and 14.3 mm were found against *P. vulgaris and E. coli*, respectively, at the same dose (90 mg/mL) of synthesized AgNPs and aqueous extract. The major phytoconstituents associated with *S. melongena* peels are glycol‐alkaloids and anthocyanins, which provide resistance by inhibiting the growth of bacterial cell walls (Ahmed et al. 2016). Researcher found that ginger peel showed greater antibacterial potential with inhibition zones of 10, 12, 10 and 11 mm against *E. coli, S*. *aureus* , *Proteus vulgaris*, and *Lactobacillus* strains, respectively (Singh et al. 2021). The promising antibacterial potential of green synthesized silver nanoparticles was due to the electrostatic binding of positively charged Ag + ions to the naked peptides on the bacterial cell walls (Salayová et al. 2021). It was observed that *B. subtilis* showed more resistance at all concentrations of aqueous extract with inhibition zones (mm) ranging from 7.3 ± 0.7 to 13.2 ± 1.3 mm at the concentrations of 30 mg/mL‐90 mg/mL. While relatively more antibacterial potential was observed by synthesized AgNPs (ZOI 9.6.3 ± 0.5–13.5 ± 1.5 mm) against *B. subtilis*. Asif et al. (2022) suggested that the toxicity of silver nanoparticles against Gram‐negative strains was due to the oxidative stress induced by independent silver ions in bacterial cells. It was observed that bacterial cells treated with green synthesized AgNPs showed accumulation of reactive oxygen species (ROS), increased intracellular calcium levels, the exposure of phosphatidylserine in the outer membrane, which indicate early apoptosis, the activation of bacterial caspase‐like proteins, degradation of DNA, disruption of membrane potential, which is the sign of late apoptosis in bacterial cells (Bedlovicová and Salayová 2017). Thus, the findings conclude that synthesized AgNPs using *S. melongena* peels with promising antimicrobial activity could be practically used to control bacterial infections.

### 3.5 Hemolytic Activity

The hemolysis assay results revealed that both the aqueous extract of *S. melongena* and its silver nanoparticles (AgNPs) induced minimal red blood cell lysis, indicating good biocompatibility (Table 4). For aqueous extract, percentage hemolysis increased gradually with concentration, from 2.34% ± 1.32% at 30 mg/mL to 4.98% ± 1.43% at 90 mg/mL. Similarly, *S. melongena* ‐mediated AgNPs exhibited a slight rise in hemolytic activity, from 2.09% ± 1.39% at 30 mg/mL to 3.98% ± 0.98% at 90 mg/mL. In contrast, the positive control, Triton X‐100 (0.1%), caused 100% hemolysis, confirming the assay's validity. Hemolytic capacity describes a substance's ability—such as that of a natural product extract—to induce hemolysis in erythrocytes or to inhibit hemolysis caused by a known hemolytic agent like hydrogen peroxide (H_2_O_2_), Triton X‐100, or 2,2′‐azobis (2‐amidinopropane) dihydrochloride (AAPH), which can reflect its antioxidant potential (Peña‐Medina et al. 2023). This evaluation is important for studying natural antioxidants and other bioactive molecules also in case of nanoparticles like AgNO_3_, ZnO and CuSO_4_ with potential therapeutic roles in conditions where hemolysis is a major concern, such as certain anemias also for assessing the safety of potentially harmful agents, including the overuse of specific treatments or toxic natural products (Busari et al. 2024). It is vital to identify the natural compounds that may serve as preventive or supportive treatments for hemolytic disorders (Quintanilla‐Licea et al. 2023). Moreover, plants and other natural products are frequently investigated for their ability to protect red blood cells from oxidative stress. Hemolysis testing is an essential step in the preclinical safety assessment of novel herbal medicines before human application. In these studies, a lower hemolysis percentage in the presence of the tested natural product indicates greater anti‐hemolytic potential (Bogucka‐Kocka et al. 2018).

**TABLE 4:** Hemolytic activity of aqueous and *S. melongena* ‐mediated AgNPs.

Sample	Concentration	Percentage hemolysis
*S. melongena* aqueous extract	30 mg/mL	2.34 ± 1.32
	60 mg/mL	3.56 ± 1.09
	90 mg/mL	4.98 ± 1.43
*S. melongena* mediated‐AgNO_3_‐NPs	30 mg/mL	2.09 ± 1.39
	60 mg/mL	3.11 ± 1.09
	90 mg/mL	3.98 ± 0.98
Triton X‐100	0.1%	100⁒

*Note:* Values are expressed as the mean ± SD.

### 3.6 Wound Healing Potential

The rabbits were treated with three different concentrations (30, 60, and 90 mg/mL) of crude and AgNPs of *S. melongena* (Table 5). On Day 7th and 14th, the animals' wound sizes were measured. The findings demonstrate a concentration‐dependent rise in wound healing. The decrease in wound size observed on the seventh day was 43% at the lowest dose (30 mg/mL) of aqueous extract in G‐3. In contrast, it was 60.6% for AgNPs in G‐6. On the 7th day, the wound reduction at the highest concentration was recorded as 49.9% and 64% for these groups, respectively. On the 14th day, wound sizes of all tested concentrations reduced significantly at the highest concentration of both extracts, 60% (aqueous extract) and 83% (AgNPs). By comparing crude extract and nanoparticle efficacy in the case of wound reduction, synthesized nanoparticles accelerate the healing process more than crude extract. Results were clearly consistent with the results of Pammi et al. (2023). The last group treated with the highest concentration of 90 mg/mL of nanocomposites shows more healing due to the start of rapid regeneration. Contraction, an important process, can be visualized in G‐8 and G‐1, which indicated a reduced wound size and hence healing time. Overall, contraction is a crucial step involved in wound healing, which reduces the amount of extracellular matrix required for re‐epithelialization and reduces the distance for migrating keratinocytes (Yazarlu et al. 2021). Not only contraction but epithelization is another important step in wound healing. The epithelization period is a time in which epithelial organization takes place, which is another crucial step in the wound healing process (Ananda et al. 2022). Because of their special qualities—small size and vast surface area, mechanical and thermal durability, chemical inertness, electrical conductivity, biosensor, and antibacterial activity—silver nanoparticles have emerged as a major player in the management of wound healing (Nqakala et al. 2021). The presence of functional groups in FTIR spectra of amine, amide, and phenolic compounds suggested that the terpenoids, alkaloids, and flavonoids in the extract acted as reducing agents when the AgNPs were being formed (Chinnasamy et al. 2021). Green synthesis of silver nanoparticles (AgNPs) using plant extracts enhances their biocompatibility by avoiding toxic chemicals and utilizing natural reducing and stabilizing agents (Singh et al. 2023). The phytochemicals in plant extracts, such as antioxidants, aid in controlled nanoparticle formation and stabilization. This eco‐friendly approach minimizes cytotoxicity and improves biological compatibility. Consequently, green‐synthesized AgNPs hold great promise for safe and effective biomedical applications (Shahzadi et al. 2025).

**TABLE 5:** Reduction in wound size by application of *Solanum melongena* extract.

Group	Group description	0‐Day	7th Day	14th Day
G‐1	Control +Ve	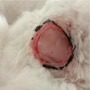	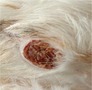	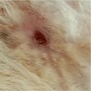
		3%–0%	2.13% ± 0.10%–71%	2.76% ± 0.07%–92%
G‐2	Control –Ve	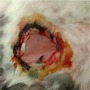	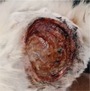	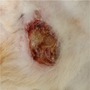
		3%–0%	0.94% ± 1.21%–31.33%	1.35% ± 0.09%–45%
G‐3	SM‐C (30 mg/mL)	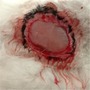	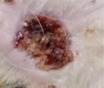	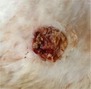
		3%–0%	1.29% ± 1.92%–43%	1.68% ± 0.26%–56%
G‐4	SM‐C (60 mg/mL)	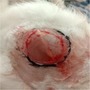	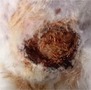	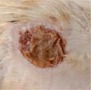
		3%–0%	1.36% ± 1.01%–45%	1.79% ± 0.31%–59%
G‐5	SM‐C (90 mg/mL)	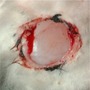	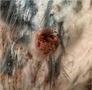	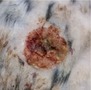
		3%–0%	1.49% ± 1.84%–49.6%	1.80% ± 0.24%–60%
G‐6	SM‐AgNPs (30 mg/mL)	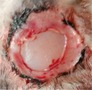	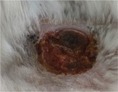	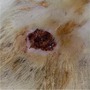
		3%–0%	1.82% ± 1.25%–60.6%	2.38% ± 0.10%–79.3%
G‐7	SM‐AgNPs (60 mg/mL)	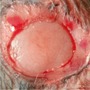	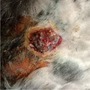	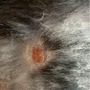
		3%–0%	1.89% ± 0.106%–63%	2.41% ± 0.16%–80%
G‐8	SM‐AgNPs (90 mg/mL)	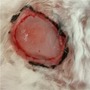	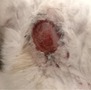	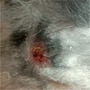
		3%–0%	1.93% ± 1.145%–64%	2.49% ± 0.18%–83%

*Note:* Values are expressed as the mean ± SD. SM‐C represents the *Solanum melongena* crude extract taken at three different concentrations: 30, 60, and 90 mg/mL. while SM‐AgNPs represent AgNPs mediated by *Solanum melongena* extract taken at 30, 60, and 90 mg/mL.

To assess statistical differences in wound healing activity among treatment groups, ANOVA was performed separately for each group. When significant differences were detected, Tukey's post hoc test was used to identify specific pairwise comparisons (Table 6). Table 7 gives the day wise comparative analysis of wound size. The analysis showed that positive control G‐1 shows the highest wound healing effectiveness with *p* value of 0.05 which is significantly different (*p* < 0.05) than any concentration of *S. melongena* extract or AgNPs. Additionally, higher concentrations of both AgNPs and *S. melongena* extract exhibited significantly greater wound healing activity compared to lower concentrations, indicating a clear dose‐dependent effect. These results highlight the relative efficacy of the treatments and emphasize the wound treating potential of AgNPs and plant‐derived extracts.

**TABLE 6:** Statistical analysis of group wise comparative analysis of wound size through Tukey's post hoc test.

(I) Group	(J) Group	Mean difference (I–J)	Std. error	Sig.	95%…
					Lower bound
	BRI‐S (90 mg/mL)	0.3383	0.56996	0.999	−1.5493
	BRI‐AgNO3 (30 mg/mL)	0.0350	0.56996	1.000	−1.8527
	BRI‐AgNO3 (90 mg/mL)	−0.0367	0.56996	1.000	−1.9243
BRI‐AgNO3 (90 mg/mL)	Control +Ve	−0.1583	0.56996	1.000	−2.0460
	Control −Ve	0.7100	0.56996	0.910	−1.1777
	BRI‐S (30 mg/mL)	0.4817	0.56996	0.988	−1.4060
	BRI‐S (60 mg/mL)	0.4217	0.56996	0.995	−1.4660
	BRI‐S (90 mg/mL)	0.3750	0.56996	0.997	−1.5127
	BRI‐AgNO3 (30 mg/mL)	0.0717	0.56996	1.000	−1.8160
	BRI‐AgNO3 (60 mg/mL)	0.0367	0.56996	1.000	−1.8510

**TABLE 7:** Day wise comparative analysis of wound size.

(I) Day	(J) Day	Mean difference (I–J)	Std. error	Sig.	95% Confidence interval	
					Lower bound	Upper bound
0	7	1.3969*	0.34903	0.001	0.5253	2.2685
	14	0.9200*	0.34903	0.037	0.0484	1.7916
7	0	−1.3969*	0.34903	0.001	−2.2685	−0.5253
	14	−0.4769	0.34903	0.374	−1.3485	0.3947
14	0	−0.9200*	0.34903	0.037	−1.7916	−0.0484
	7	0.4769	0.34903	0.374	−0.3947	1.3485

*Note:* Based on observed means. The error term is Mean Square (Error) = 0.975.

* The mean difference is significant at the 0.05 level.

### 3.7 Histopathology Studies

**FIGURE 11:**
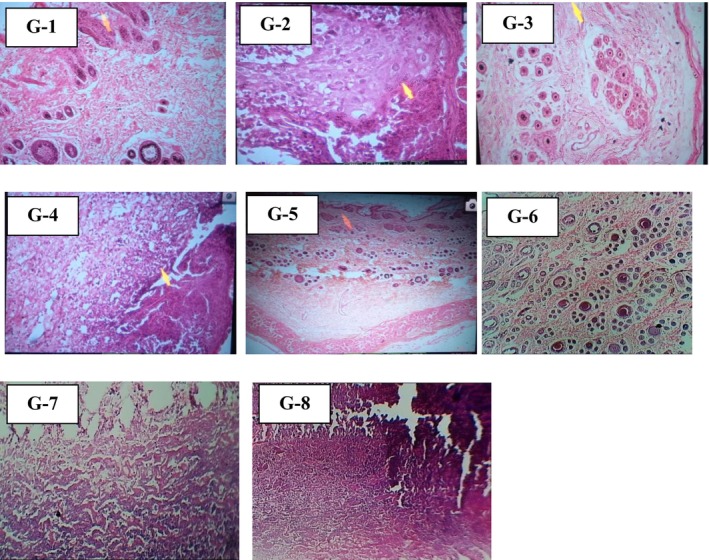
Histopathological evaluation of wound repairing capacity of *Solanum melongena* extract.

A histopathological study of the collected sample on the 7th day of the experiment showed a slight difference between the positive control group and the nanoparticle‐treated group and is slightly different from the crude‐treated group, as the tissues showed mild angiogenesis and infiltration of a heterogeneous population of inflammatory cells, predominantly neutrophils (Table 8) visually inspected from Figure 11. It indicates an inflammatory phase, characterized by the formation of a small amount of loosely arranged immature granulation tissue (Zhou et al. 2018). Groups treated with varying concentrations of crude and AgNPs extracts showed differences in efficiency of healing and regeneration as compared to control groups; angiogenesis and epithelial cell proliferation at the wound edges were prominent. The group treated with 90 mg/mL of extract showed marked epithelial hyperplasia, angiogenesis, fibroblast proliferation, and replacement of neutrophils with macrophages. *S. melongena* peel has antibacterial properties due to the presence of phenolics and shows considerable therapeutic efficiency in wound healing (Khorasani et al. 2018). The wound area demonstrated epithelial regeneration, mononuclear cell infiltration, blood vessel formation, fibroblast proliferation, a mild degree of extracellular matrix deposition, and clear visibility of hair follicles when different concentrations of crude extracts carried the treatments, according to the results. Even on the 7th day of treatment, blood vessels are positioned perpendicular to the wound surface, evident collagen deposition is induced, and comparatively few mononuclear inflammatory cells are present in the collagen deposition. It results from the administration of *Solanum melongena* AgNPs. The highest level of epithelial regeneration was seen in G‐8, suggesting that the bioactive capped AgNPs (90 mg/mL) ointment was a useful treatment for wound healing. In contrast, wound sites in G‐5 still have a low level of inflammation despite dermal granulation as compared to Group‐8.

**TABLE 8:** Histopathological reviews of collected skin samples.

Group. no	Group description	Histopathological review
G‐1	Positive control	Fibroblasts proliferation, moderate inflammation and deposition of extracellular matrix beneath the scabby tissue are present. No epithelial regeneration is visible.
G‐2	Negative control	High inflammation, neutrophils accumulation and abscess formation with little concentration of pus.
G‐3	SM‐C (30 mg/mL)	Thickened epidermis, collagen fibers denser and more visible and more hair follicles than control
G‐4	SM‐C (60 mg/mL)	Inflammatory phase seen; plasma cells seen along with tissue debris.
G‐5	SM‐C (90 mg/mL)	Circumscribed area looks granulation comprised of neutrophils newly formed capillaries and fibroblasts
G‐6	SM‐AgNPs (30 mg/mL)	Excellent granulation mass
G‐7	SM‐AgNPs (60 mg/mL)	Although evident, epithelial regeneration is still ongoing. Granulation tissue and a moderate infiltration of mononuclear inflammatory cells are visible in the dermis.
G‐8	SM‐AgNPs (90 mg/mL)	Maximal renewal of the epithelium in the dermis, there are comparatively few mononuclear inflammatory cells along with collagen deposition and fibroblastic growth.

*Note:* Here SM‐C represents the *Solanum melongena* crude extract taken at three different concentrations: 30, 60, and 90 mg/mL. while SM‐AgNPs represent AgNPs mediated by *Solanum melongena* extract taken at 30, 60, and 90 mg/mL.

## 4 Conclusions

This comparative study demonstrates the wound‐healing efficacy of aqueous and AgNPs‐synthesized *Solanum melongena* extracts. Green‐synthesized silver nanoparticles (AgNPs) using plant extracts exhibit enhanced biocompatibility due to natural reducing agents that minimize toxicity. This eco‐friendly approach ensures safer and more effective biomedical applications. The extract's therapeutic activity is attributed to its rich nutritional value. Phytochemical analysis confirmed substantial TPC and TFC, and LC–MS identified the key phytocompounds campestral, cycloeucalenone, and neochlorogenic acid. Antioxidant activity by DPPH assay reached 68.67% for the aqueous extract and 81.87% for the *S. melongena* mediated silver nanoparticle. The antioxidant enzyme activity revealed 31.49 IU/mL (CAT), 15.92 IU/mL (SOD), and 10.37 IU/mL (POD). AgNPs displayed broad‐spectrum antibacterial activity, with a maximum ZOI of 19.5 mm against *Bacillus subtilis* . The hemolytic analysis revealed its safety profile. Physicochemical characterization (FTIR, SEM, XRD) verified the functional groups and nanoparticle morphology; AgNPs diameter measured 30–52 nm with an average crystalline size of 15.37 nm. In vivo wound healing potential results showed that AgNPs result in higher wound‐size reduction than crude extract, accompanied by reduced inflammation and enhanced fibroblast proliferation, angiogenesis, collagen deposition, and re‐epithelialization. These results position the AgNP‐formulated extract as a promising delivery platform for accelerated wound healing, necessitating further mechanistic and safety evaluation.

## Author Contributions

**Dure Shahwar:** investigation, data curation, methodology, statistical analysis, writing original draft; **Mazhar Abbas:** conceptualization, project administration, supervision, resources, validation; **Kinza Zafar, Waqas Haider, Maha Gul Zafar**, and **Muhammad Haseeb Zafar:** investigation, methodology, analysis, validation, writing original draft; **Tariq Hussain:** validation, visualization, review and editing; **Fozia Anjum, Ghulam Rasool** and **Muhammad Riaz:** analysis, validation, visualization, review and editing; **Hasan Ejaz** and **Quzi Sharmin Akter:** analysis, visualization, writing – review editing.

## Funding

The authors have nothing to report.

## Ethics Statement

The study plan was approved by the ethical committee of the College of Veterinary and Animal Science, Jhang, Pakistan.

## Conflicts of Interest

The authors declare no conflicts of interest.

## Data Availability

Data will be available from principal and corresponding authors on reasonable request.

## References

[fsn371569-bib-0001] Abbas, M. , A. Ali , M. Arshad , et al. 2018. “Mutagenicity, Cytotoxic and Antioxidant Activities of *Ricinus communis* Different Parts.” Chemistry Central Journal 12, no. 1: 3. 29350299 10.1186/s13065-018-0370-0 PMC5775190

[fsn371569-bib-0002] Abbas, M. , M. Arshad , M. Rafique , et al. 2022. “Chitosan‐Polyvinyl Alcohol Membranes With Improved Antibacterial Properties Contained *Calotropis procera* Extract as a Robust Wound Healing Agent.” Arabian Journal of Chemistry 15, no. 5: 103766.

[fsn371569-bib-0003] Abbas, M. , A. Mumtaz , O. A. Mohammed , et al. 2025. “Green‐Synthesized ZnO Nanoparticles Using Methanolic Extract of *Moringa oleifera* Leaves: Characterization and Evaluation of Wound Healing Efficacy.” Journal of Pharmaceutical Innovation 20, no. 4: 112.

[fsn371569-bib-0004] Afreen, A. , R. Ahmed , S. Mehboob , et al. 2020. “Phytochemical‐Assisted Biosynthesis of Silver Nanoparticles From Ajuga Bracteosa for Biomedical Applications.” Materials Research Express 7, no. 7: 75404.

[fsn371569-bib-0005] Aftab, J. , M. Abbas , S. Sharif , et al. 2024. “LC‐MS/MS Profiling, Antioxidant Potential and Cytotoxicity Evaluation of *Citrus reticulata* Albedo.” Natural Product Communications 19, no. 8: 72471.

[fsn371569-bib-0006] Ahmed, F. A. , S. Mubassara , and T. Sultana . 2016. “Phytoconstituents, Bioactivity and Antioxidant Potential of Some Commercial Brinjal (*Solanum melongena* L.) Cultivars of Bangladesh.” Jahangirnagar University Journal of Biological Sciences 5, no. 2: 41–50.

[fsn371569-bib-0007] Amponsah, I. K. , A. Boakye , E. Orman , et al. 2022. “Assessment of Some Quality Parameters and Chemometric‐Assisted FTIR Spectral Analysis of Commercial Powdered Ginger Products on the Ghanaian Market.” Heliyon 8, no. 3: 9150. 10.1016/j.heliyon.2022.e09150 PMC9280518 35846447

[fsn371569-bib-0008] Ananda, N. , D. Ariawan , and V. Juniantito . 2022. “Effects of the *Hydnophytum formicarum* Plant Extract on Collagen Density, Angiogenesis, Wound Length, and Re‐Epithelialization in Wound Healing: Experimental Study on Rats.” Dental and Medical Problems 59, no. 1: 67–73. 35274499 10.17219/dmp/140208

[fsn371569-bib-0009] Anibarro‐Ortega, M. , M. I. Dias , J. Petrović , et al. 2025. “Valorization of *Solanum melongena* L. Crop By‐Products: Phenolic Composition and In Vitro Antioxidant, Antidiabetic, Anti‐Inflammatory, Cytotoxic, and Antimicrobial Properties.” Process Biochemistry 153: 315–324.

[fsn371569-bib-0010] Asif, M. , R. Yasmin , R. Asif , A. Ambreen , M. Mustafa , and S. Umbreen . 2022. “Green Synthesis of Silver Nanoparticles (AgNPs), Structural Characterization, and Their Antibacterial Potential.” Dose‐Response 20, no. 2: 8709. 10.1177/15593258221088709 PMC9112420 35592270

[fsn371569-bib-0011] Bedlovicová, Z. , and A. Salayová . 2017. “Green‐Synthesized Silver Nanoparticles and Their Potential for Antibacterial Applications.” Bacterial Pathogenesis and Antibacterial Control.

[fsn371569-bib-0012] Bogucka‐Kocka, A. , C. Zidorn , M. Kasprzycka , G. Szymczak , and K. Szewczyk . 2018. “Phenolic Acid Content, Antioxidant and Cytotoxic Activities of Four Kalanchoë Species.” Saudi Journal of Biological Sciences 25, no. 4: 622–630. 29740226 10.1016/j.sjbs.2016.01.037 PMC5936878

[fsn371569-bib-0013] Boopathi, S. , S. Gopinath , T. Boopathi , V. Balamurugan , R. Rajeshkumar , and M. Sundararaman . 2012. “Characterization and Antimicrobial Properties of Silver and Silver Oxide Nanoparticles Synthesized by Cell‐Free Extract of a Mangrove‐Associated *Pseudomonas aeruginosa* M6 Using Two Different Thermal Treatments.” Industrial & Engineering Chemistry Research 51, no. 17: 5976–5985.

[fsn371569-bib-0014] Bullock, C. J. , and C. Bussy . 2019. “Biocompatibility Considerations in the Design of Graphene Biomedical Materials.” Advanced Materials Interfaces 6, no. 11: 1900229.

[fsn371569-bib-0015] Busari, I. O. , J. H. Elizondo‐Luévano , O. O. Aiyelaagbe , et al. 2024. “Anthelmintic Activity of Three Selected Ethnobotanical Plant Extracts Against Strongyloides Venezuelensis.” Experimental Parasitology 263: 108801. 39009180 10.1016/j.exppara.2024.108801

[fsn371569-bib-0016] Cao, Z. , Z. Wang , Z. Shang , and J. Zhao . 2017. “Classification and Identification of *Rhodobryum roseum* Limpr. And Its Adulterants Based on Fourier‐Transform Infrared Spectroscopy (FTIR) and Chemometrics.” PLoS One 12, no. 2: e0172359. 28207900 10.1371/journal.pone.0172359 PMC5313229

[fsn371569-bib-0017] Carneiro, J. d. S. , R. M. Nogueira , M. A. Martins , D. M. d. S. Valladão , and E. M. Pires . 2018. “The Oven‐Drying Method for Determination of Water Content in Brazil Nut.”

[fsn371569-bib-0018] Che, J. , T. Zhao , W. Liu , et al. 2021. “Neochlorogenic Acid Enhances the Antitumor Effects of Pingyangmycin via Regulating TOP2A.” Molecular Medicine Reports 23, no. 2: 1. 10.3892/mmr.2020.11797 33355372

[fsn371569-bib-0019] Chinedu, S. N. , A. C. Olasumbo , O. K. Eboji , O. C. Emiloju , O. K. Arinola , and D. I. Dania . 2011. “Proximate and Phytochemical Analyses of *Solanum aethiopicum* L. and *Solanum macrocarpon* L. Fruits.” Research Journal of Chemical Sciences 1, no. 3: 63–71.

[fsn371569-bib-0020] Chinnasamy, G. , S. Chandrasekharan , T. W. Koh , and S. Bhatnagar . 2021. “Synthesis, Characterization, Antibacterial and Wound Healing Efficacy of Silver Nanoparticles From *Azadirachta indica* .” Frontiers in Microbiology 12: 611560. 33679635 10.3389/fmicb.2021.611560 PMC7932996

[fsn371569-bib-0021] Choudhury, K. , D. Sarma , P. J. Sapruna , and A. D. Soren . 2020. “Proximate and Mineral Compositions of *Samia cynthia* Ricini and *Dytiscus marginalis*, Commonly Consumed by the Bodo Tribe in Assam, India.” Bulletin of the National Research Centre 44, no. 1: 1–7.

[fsn371569-bib-0022] Comino‐Sanz, I. M. , M. D. López‐Franco , B. Castro , and P. L. Pancorbo‐Hidalgo . 2021. “The Role of Antioxidants on Wound Healing: A Review of the Current Evidence.” Journal of Clinical Medicine 10, no. 16: 3558. 34441854 10.3390/jcm10163558 PMC8397081

[fsn371569-bib-0023] Dadgostar, P. 2019. “Antimicrobial Resistance: Implications and Costs.” Infection and Drug Resistance 12: 3903–3910. 31908502 10.2147/IDR.S234610 PMC6929930

[fsn371569-bib-0024] Das, G. , J. K. Patra , T. Debnath , A. Ansari , and H.‐S. Shin . 2019. “Investigation of Antioxidant, Antibacterial, Antidiabetic, and Cytotoxicity Potential of Silver Nanoparticles Synthesized Using the Outer Peel Extract of *Ananas comosus* (L.).” PLoS One 14, no. 8: e0220950. 31404086 10.1371/journal.pone.0220950 PMC6690543

[fsn371569-bib-0025] Das, R. K. , and D. Bhuyan . 2019. “Microwave‐Mediated Green Synthesis of Gold and Silver Nanoparticles From Fruit Peel Aqueous Extract of *Solanum melongena* L. and Study of Antimicrobial Property of Silver Nanoparticles.” Nanotechnology for Environmental Engineering 4, no. 1: 5.

[fsn371569-bib-0026] Das, S. , L. Langbang , M. Haque , V. K. Belwal , K. Aguan , and A. S. Roy . 2021. “Biocompatible Silver Nanoparticles: An Investigation Into Their Protein Binding Efficacies, Anti‐Bacterial Effects and Cell Cytotoxicity Studies.” Journal of Pharmaceutical Analysis 11, no. 4: 422–434. 34513118 10.1016/j.jpha.2020.12.003 PMC8424387

[fsn371569-bib-0027] David, A. V. A. , R. Arulmoli , and S. Parasuraman . 2016. “Overviews of Biological Importance of Quercetin: A Bioactive Flavonoid.” Pharmacognosy Reviews 10, no. 20: 84. 28082789 10.4103/0973-7847.194044 PMC5214562

[fsn371569-bib-0028] Devi, L. S. , and S. Joshi . 2015. “Ultrastructures of Silver Nanoparticles Biosynthesized Using Endophytic Fungi.” Journal of Microscopy and Ultrastructure 3, no. 1: 29–37. 30023179 10.1016/j.jmau.2014.10.004 PMC6014216

[fsn371569-bib-0029] Djouadi, A. , T. Lanez , and C. Boubekri . 2016. “Evaluation of Antioxidant Activity and Polyphenolic Contents of Two South Algerian Eggplants Cultivars.” Journal of Fundamental and Applied Sciences 8, no. 2: 223–231.

[fsn371569-bib-0030] Duan, H. , D. Wang , and Y. Li . 2015. “Green Chemistry for Nanoparticle Synthesis.” Chemical Society Reviews 44, no. 16: 5778–5792. 25615873 10.1039/c4cs00363b

[fsn371569-bib-0031] Dulta, K. , G. Koşarsoy Ağçeli , P. Chauhan , R. Jasrotia , and P. Chauhan . 2022. “Ecofriendly Synthesis of Zinc Oxide Nanoparticles by *Carica papaya* Leaf Extract and Their Applications.” Journal of Cluster Science 33, no. 2: 603–617.

[fsn371569-bib-0032] Dumanović, J. , E. Nepovimova , M. Natić , K. Kuča , and V. Jaćević . 2021. “The Significance of Reactive Oxygen Species and Antioxidant Defense System in Plants: A Concise Overview.” Frontiers in Plant Science 11: 552969. 33488637 10.3389/fpls.2020.552969 PMC7815643

[fsn371569-bib-0033] Egbuonu, A. C. C. , and C. A. Osuji . 2016. “Proximate Compositions and Antibacterial Activity of *Citrus sinensis* (Sweet Orange) Peel and Seed Extracts.” European Journal of Medicinal Plants 12, no. 3: 1–7.

[fsn371569-bib-0034] Espíndola, K. M. M. , R. G. Ferreira , L. E. M. Narvaez , et al. 2019. “Chemical and Pharmacological Aspects of Caffeic Acid and Its Activity in Hepatocarcinoma.” Frontiers in Oncology 9: 541. 31293975 10.3389/fonc.2019.00541 PMC6598430

[fsn371569-bib-0035] Estrella‐Osuna, D. E. , J. A. Tapia‐Hernández , S. Ruíz‐Cruz , et al. 2022. “Nanoencapsulation of Eggplant (*Solanum melongena* L.) Peel Extract in Electrospun Gelatin Nanofiber: Preparation, Characterization, and In Vitro Release.” Nanomaterials 12, no. 13: 2303. 35808139 10.3390/nano12132303 PMC9268290

[fsn371569-bib-0201] Fadlelmoula, A. , D. Pinho , V. H. Carvalho , S. O. Catarino , and G. Minas . 2022. “Fourier Transform Infrared (FTIR) Spectroscopy to Analyse Human Blood over the Last 20 Years: A Review Towards Lab‐on‐a‐Chip Devices.” Micromachines 13, no. 2: 187. 35208311 10.3390/mi13020187 PMC8879834

[fsn371569-bib-0036] Fujita, M. , and M. Hasanuzzaman . 2022. Approaches to Enhancing Antioxidant Defense in Plants. Vol. 11, 925. MDPI. 10.3390/antiox11050925 PMC9137904 35624789

[fsn371569-bib-0037] Gallo, M. , D. Naviglio , and L. Ferrara . 2014. “Nasunin, an Antioxidant Anthocyanin From Eggplant Peels, as Natural Dye to Avoid Food Allergies and Intolerances.” European Scientific Journal 10, no. 9: 819.

[fsn371569-bib-0038] Gao, J. , J. Hu , D. Hu , and X. Yang . 2019. “A Role of Gallic Acid in Oxidative Damage Diseases: A Comprehensive Review.” Natural Product Communications 14, no. 8: 4174.

[fsn371569-bib-0039] Geoprincy, G. , B. V. Srri , U. Poonguzhali , N. N. Gandhi , and S. Renganathan . 2013. “A Review on Green Synthesis of Silver Nanoparticles.” Asian Journal of Pharmaceutical and Clinical Research 6, no. 1: 8–12.

[fsn371569-bib-0040] Hazra, P. 2023. Antioxidants and Health Benefits of Brinjal Vegetables for Nutrition and Entrepreneurship, 203–216. Springer.

[fsn371569-bib-0041] Hewavitharana, G. G. , D. N. Perera , S. Navaratne , and I. Wickramasinghe . 2020. “Extraction Methods of Fat From Food Samples and Preparation of Fatty Acid Methyl Esters for Gas Chromatography: A Review.” Arabian Journal of Chemistry 13, no. 8: 6865–6875.

[fsn371569-bib-0042] Hossain, M. E. , S. A. Sultana , M. H. Karim , and M. I. Ahmed . 2015. “Vegetable Peels: A Promising Feed Resource for Livestock.”

[fsn371569-bib-0043] Hussain, T. , D. H. Kalhoro , and Y. Yin . 2023. “Identification of Nutritional Composition and Antioxidant Activities of Fruit Peels as a Potential Source of Nutraceuticals.” Frontiers in Nutrition 9: 1065698. 36817065 10.3389/fnut.2022.1065698 PMC9931757

[fsn371569-bib-0044] Jangid, H. , S. Singh , P. Kashyap , A. Singh , and G. Kumar . 2024. “Advancing Biomedical Applications: An In‐Depth Analysis of Silver Nanoparticles in Antimicrobial, Anticancer, and Wound Healing Roles.” Frontiers in Pharmacology 15: 1438227. 39175537 10.3389/fphar.2024.1438227 PMC11338803

[fsn371569-bib-0045] Jantas, D. , J. Chwastek , J. Malarz , A. Stojakowska , and W. Lasoń . 2020. “Neuroprotective Effects of Methyl Caffeate Against Hydrogen Peroxide‐Induced Cell Damage: Involvement of Caspase 3 and Cathepsin D Inhibition.” Biomolecules 10, no. 11: 1530. 33182454 10.3390/biom10111530 PMC7696984

[fsn371569-bib-0046] Jemal, K. , B. Sandeep , and S. Pola . 2017. “Synthesis, Characterization, and Evaluation of the Antibacterial Activity of Allophylus Serratus Leaf and Leaf Derived Callus Extracts Mediated Silver Nanoparticles.” Journal of Nanomaterials 2017, no. 1: 4213275.

[fsn371569-bib-0047] Kainat, F. , M. Ali , A. Akbar , R. Masih , S. Mehnaz , and M. B. Sadiq . 2023. “Ultrasonic Extraction of Phenolic Compounds From Eggplant Peel and Formulation of Eggplant Peel Extract‐Enriched Ice‐Cream.” Journal of Food Quality 2023, no. 1: 3267119.

[fsn371569-bib-0048] Kaiser, P. , J. Wächter , and M. Windbergs . 2021. “Therapy of Infected Wounds: Overcoming Clinical Challenges by Advanced Drug Delivery Systems.” Drug Delivery and Translational Research 11, no. 4: 1545–1567. 33611768 10.1007/s13346-021-00932-7 PMC8236057

[fsn371569-bib-0049] Kalloo, G. , and B. Bergh . 2012. Genetic Improvement of Vegetable Crops. Newnes.

[fsn371569-bib-0050] Kellab, R. , F. Boulkenafet , S. Amokrane , et al. 2024. “Chemical Profiling and In Vitro Evaluation of the Antioxidant, Anti‐Inflammatory, and Anti‐Bacterial Effects of Algerian *Solanum melongena* L.” Indian Journal of Pharmaceutical Education And Research 59, no. 1: 338–350.

[fsn371569-bib-0204] Khorasani, S. , M. Danaei , and M. R. Mozafari . 2018. “Nanolipo Some Technology for the Food and Nutraceutical Industries.” Trends in Food Science & Technology 79: 106–115.

[fsn371569-bib-0202] Koga, D. , S. Kusumi , M. Shibata , and T. Watanabe . 2021. “Applications of Scanning Electron Microscopy Using Secondary and Backscattered electron Signals in Neural Structure.” Frontiers in Neuroanatomy 15: 759804. 34955763 10.3389/fnana.2021.759804 PMC8693767

[fsn371569-bib-0051] Kolimi, P. , S. Narala , D. Nyavanandi , A. A. A. Youssef , and N. Dudhipala . 2022. “Innovative Treatment Strategies to Accelerate Wound Healing: Trajectory and Recent Advancements.” Cells 11, no. 15: 2439. 35954282 10.3390/cells11152439 PMC9367945

[fsn371569-bib-0052] Kona, N. , M. B. Islam , J. Alam , et al. 2024. “Antiproliferative, Pharmacological and Antibacterial Activities of Synthesized Silver Nanoparticles From the Combined Extract of *Pandanus foetidus* and *Alangium salvifolium* .” Results in Chemistry 7: 101547.

[fsn371569-bib-0053] Kongkathip, N. , P. Dhumma‐upakorn , B. Kongkathip , K. Chawananoraset , P. Sangchomkaeo , and S. Hatthakitpanichakul . 2002. “Study on Cardiac Contractility of Cycloeucalenol and Cycloeucalenone Isolated From Tinospora Crispa.” Journal of Ethnopharmacology 83, no. 1–2: 95–99. 12413712 10.1016/s0378-8741(02)00210-6

[fsn371569-bib-0054] Krishnaraj, C. , E. Jagan , S. Rajasekar , P. Selvakumar , P. Kalaichelvan , and N. Mohan . 2010. “Synthesis of Silver Nanoparticles Using *Acalypha indica* Leaf Extracts and Its Antibacterial Activity Against Water Borne Pathogens.” Colloids and Surfaces B: Biointerfaces 76, no. 1: 50–56. 19896347 10.1016/j.colsurfb.2009.10.008

[fsn371569-bib-0055] Kumar, R. , S. Dutt , A. D. Tripathi , A. K. Singh , V. K. Chaturvedi , and S. K. Singh . 2024. “Navigating Safety and Toxicity Challenges in Nanomedicine: Strategies, Assessment, and Mitigation.” In Nanomedicine: Innovations, Applications, and Breakthroughs in the Quest for Health and Medicine's Future, 15–37. Springer.

[fsn371569-bib-0056] Li, X. , Y. Tian , T. Wang , et al. 2017. “Role of the p‐Coumaroyl Moiety in the Antioxidant and Cytoprotective Effects of Flavonoid Glycosides: Comparison of Astragalin and Tiliroside.” Molecules 22, no. 7: 1165. 28704976 10.3390/molecules22071165 PMC6152332

[fsn371569-bib-0057] Logeswari, P. , S. Silambarasan , and J. Abraham . 2015. “Synthesis of Silver Nanoparticles Using Plants Extract and Analysis of Their Antimicrobial Property.” Journal of Saudi Chemical Society 19, no. 3: 311–317.

[fsn371569-bib-0058] Mat Yusuf, S. N. A. , C. N. A. Che Mood , N. H. Ahmad , D. Sandai , C. K. Lee , and V. Lim . 2020. “Optimization of Biogenic Synthesis of Silver Nanoparticles From Flavonoid‐Rich Clinacanthus Nutans Leaf and Stem Aqueous Extracts.” Royal Society Open Science 7, no. 7: 200065. 32874618 10.1098/rsos.200065 PMC7428249

[fsn371569-bib-0059] Mohamed, M. , A. Zeitoun , and A. E. Abdalla . 2019. “Assessment of Chemical Composition and Bioactive Compounds in the Peel, Pulp and Whole Egyptian Eggplant Flour.” Journal of the Advances in Agricultural Researches 24, no. 1: 14–37.

[fsn371569-bib-0060] Mohan, M. , D. Attarde , R. Momin , and S. Kasture . 2013. “Antidepressant, Anxiolytic and Adaptogenic Activity of Torvanol A: An Isoflavonoid From Seeds of *Solanum torvum* .” Natural Product Research 27, no. 22: 2140–2143. 23521182 10.1080/14786419.2013.778853

[fsn371569-bib-0061] Morais, D. R. , E. M. Rotta , S. C. Sargi , et al. 2017. “Proximate Composition, Mineral Contents and Fatty Acid Composition of the Different Parts and Dried Peels of Tropical Fruits Cultivated in Brazil.” Journal of the Brazilian Chemical Society 28, no. 2: 308–318.

[fsn371569-bib-0062] Mullen, W. , J. McGinn , M. E. Lean , et al. 2002. “Ellagitannins, Flavonoids, and Other Phenolics in Red Raspberries and Their Contribution to Antioxidant Capacity and Vasorelaxation Properties.” Journal of Agricultural and Food Chemistry 50, no. 18: 5191–5196. 12188628 10.1021/jf020140n

[fsn371569-bib-0063] Naganthran, A. , G. Verasoundarapandian , F. E. Khalid , et al. 2022. “Synthesis, Characterization and Biomedical Application of Silver Nanoparticles.” Materials 15, no. 2: 427. 35057145 10.3390/ma15020427 PMC8779869

[fsn371569-bib-0064] Nayanathara, A. , A. Mathews , K. Aalolam , and J. Reshma . 2016. “Evaluation of Total Phenol, Flavonoid and Anthocyanin Content in Different Varieties of Eggplant.” Emergent Life Sciences Research 2: 63–65.

[fsn371569-bib-0065] Naz, H. , N. A. Akram , M. Ashraf , D. I. Hefft , and B. L. Jan . 2022. “Leaf Extract of Neem (*Azadirachta indica*) Alleviates Adverse Effects of Drought in Quinoa (*Chenopodium quinoa* Willd.) Plants Through Alterations in Biochemical Attributes and Antioxidants.” Saudi Journal of Biological Sciences 29, no. 3: 1367–1374. 35280556 10.1016/j.sjbs.2022.01.038 PMC8913546

[fsn371569-bib-0066] Nazir, S. , W. A. Chaudhary , A. Mobashar , I. Anjum , S. Hameed , and S. Azhar . 2023. “Campesterol: A Natural Phytochemical With Anti Inflammatory Properties as Potential Therapeutic Agent for Rheumatoid Arthritis: A Systematic Review: Campesterol: A Natural Phytochemical.” Pakistan Journal of Health Sciences 4: 792.

[fsn371569-bib-0067] Negut, I. , G. Dorcioman , and V. Grumezescu . 2020. “Scaffolds for Wound Healing Applications.” Polymers 12, no. 9: 2010. 32899245 10.3390/polym12092010 PMC7563417

[fsn371569-bib-0068] Nemzer, B. V. , D. Kalita , A. Y. Yashin , and Y. I. Yashin . 2020. “Bioactive Compounds, Antioxidant Activities, and Health Beneficial Effects of Selected Commercial Berry Fruits: A Review.” Journal of Food Research 9, no. 5: 78–101.

[fsn371569-bib-0069] Nethi, S. K. , S. Das , C. R. Patra , and S. Mukherjee . 2019. “Recent Advances in Inorganic Nanomaterials for Wound‐Healing Applications.” Biomaterials Science 7, no. 7: 2652–2674. 31094374 10.1039/c9bm00423h

[fsn371569-bib-0070] Nqakala, Z. B. , N. R. Sibuyi , A. O. Fadaka , M. Meyer , M. O. Onani , and A. M. Madiehe . 2021. “Advances in Nanotechnology Towards Development of Silver Nanoparticle‐Based Wound‐Healing Agents.” International Journal of Molecular Sciences 22, no. 20: 11272. 34681930 10.3390/ijms222011272 PMC8539597

[fsn371569-bib-0071] Pammi, S. , V. S. Padavala , T. S. K. Karumuri , et al. 2023. “Wound Healing Synergy in Wistar Albino Rats via Green Synthesized Nanoparticles and Topical Antibiotic Neomycin.” OpenNano 11: 100135.

[fsn371569-bib-0072] Peña‐Medina, R. L. , D. Fimbres‐Olivarría , L. F. Enríquez‐Ocaña , et al. 2023. “Erythroprotective Potential of Phycobiliproteins Extracted From *Porphyridium cruentum* .” Metabolites 13, no. 3: 366. 36984806 10.3390/metabo13030366 PMC10057957

[fsn371569-bib-0073] Pushparaj, K. , B. Balasubramanian , Y. Kandasamy , et al. 2023. “Green Synthesis, Characterization of Silver Nanoparticles Using Aqueous Leaf Extracts of *Solanum melongena* and In Vitro Evaluation of Antibacterial, Pesticidal and Anticancer Activity in Human MDA‐MB‐231 Breast Cancer Cell Lines.” Journal of King Saud University, Science 35, no. 5: 102663.

[fsn371569-bib-0074] Quintanilla‐Licea, R. , N. E. Rodríguez‐Garza , Á. D. Torres‐Hernández , M. J. Verde‐Star , and J. H. Elizondo‐Luévano . 2023. “Actividad Citotóxica, Antioxidante y Antihemolítica del Extracto Metanólico de Cymbopogon Citratus (DC.) Stapf.” Investigación y Desarrollo en Ciencia y Tecnología de Alimentos 8, no. 1: 957–964.

[fsn371569-bib-0075] Rahim, N. A. , M. N. F. Roslan , M. Muhamad , and A. Seeni . 2022. “Antioxidant Activity, Total Phenolic and Flavonoid Content and LC–MS Profiling of Leaves Extracts of Alstonia Angustiloba.” Separations 9, no. 9: 234.

[fsn371569-bib-0076] Ren, R. , C. Lim , S. Li , et al. 2022. “Recent Advances in the Development of Lipid‐, Metal‐, Carbon‐, and Polymer‐Based Nanomaterials for Antibacterial Applications.” Nanomaterials 12, no. 21: 3855. 36364631 10.3390/nano12213855 PMC9658259

[fsn371569-bib-0077] Riaz, M. , R. Saqlain , S. Hussain , M. Afzal , and R. Qadir . 2021. “Antidiabetic, Thrombolytic, Antimicrobial, Antioxidant and Cytotoxicity Studies of Brinjal (*Solanum melongena*) Leaves Extracts.” Lahore Garrison University Journal of Life Sciences 5, no. 2: 76–88.

[fsn371569-bib-0078] Romelle, F. D. , A. Rani , and R. S. Manohar . 2016. “Chemical Composition of Some Selected Fruit Peels.” European Journal of Food Science and Technology 4, no. 4: 12–21.

[fsn371569-bib-0079] Roumani, M. , R. E. Duval , A. Ropars , A. Risler , C. Robin , and R. Larbat . 2020. “Phenolamides: Plant Specialized Metabolites With a Wide Range of Promising Pharmacological and Health‐Promoting Interests.” Biomedicine & Pharmacotherapy 131: 110762. 33152925 10.1016/j.biopha.2020.110762

[fsn371569-bib-0080] Salayová, A. , Z. Bedlovičová , N. Daneu , et al. 2021. “Green Synthesis of Silver Nanoparticles With Antibacterial Activity Using Various Medicinal Plant Extracts: Morphology and Antibacterial Efficacy.” Nanomaterials 11, no. 4: 1005. 33919801 10.3390/nano11041005 PMC8070782

[fsn371569-bib-0081] Sathiyaseelan, A. , K. Saravanakumar , A. V. A. Mariadoss , and M.‐H. Wang . 2020. “Biocompatible Fungal Chitosan Encapsulated Phytogenic Silver Nanoparticles Enhanced Antidiabetic, Antioxidant and Antibacterial Activity.” International Journal of Biological Macromolecules 153: 63–71. 32112842 10.1016/j.ijbiomac.2020.02.291

[fsn371569-bib-0082] Scorsatto, M. , A. d. C. Pimentel , A. J. R. d. Silva , K. Sabally , G. Rosa , and G. M. M. d. Oliveira . 2017. “Assessment of Bioactive Compounds, Physicochemical Composition, and In Vitro Antioxidant Activity of Eggplant Flour.” International Journal of Cardiovascular Sciences 30: 235–242.

[fsn371569-bib-0083] Selvan, D. A. , D. Mahendiran , R. S. Kumar , and A. K. Rahiman . 2018. “Garlic, Green Tea and Turmeric Extracts‐Mediated Green Synthesis of Silver Nanoparticles: Phytochemical, Antioxidant and In Vitro Cytotoxicity Studies.” Journal of Photochemistry and Photobiology, B: Biology 180: 243–252. 29476965 10.1016/j.jphotobiol.2018.02.014

[fsn371569-bib-0084] Shahzadi, S. , S. Fatima , Z. Shafiq , and M. R. S. A. Janjua . 2025. “A Review on Green Synthesis of Silver Nanoparticles (SNPs) Using Plant Extracts: A Multifaceted Approach in Photocatalysis, Environmental Remediation, and Biomedicine.” RSC Advances 15, no. 5: 3858–3903. 39917042 10.1039/d4ra07519f PMC11800103

[fsn371569-bib-0085] Shaik, M. R. , M. Khan , M. Kuniyil , A. Al‐Warthan , H. Z. Alkhathlan , and M. R. H. Siddiqui . 2018. “Plant‐Extract‐Assisted Green Synthesis of Silver Nanoparticles Using *Origanum vulgare* L. Extract and Their Microbicidal Activities.” Sustainability 10, no. 4: 913.

[fsn371569-bib-0086] Sharma, A. , S. Khanna , G. Kaur , and I. Singh . 2021. “Medicinal Plants and Their Components for Wound Healing Applications.” Future Journal of Pharmaceutical Sciences 7, no. 1: 1–13.

[fsn371569-bib-0088] Sharma, H. , N. Chawla , and A. S. Dhatt . 2019. “Nutraceutical Content and Free Radical Scavenging Capacity of Brinjal (*Solanum melongena* L.) Genotypes.” Scientia Horticulturae 244: 294–303.

[fsn371569-bib-0087] Sharma, H. , N. Chawla , A. S. Dhatt , D. Deka , and S. Singh . 2025. “In Vitro and In Silico Study of Eggplant Extract on Human Cancer Cell Lines.” Natural Product Research 39, no. 17: 5081–5085. 38775340 10.1080/14786419.2024.2357665

[fsn371569-bib-0089] Sharma, K. , S. Kaushik , and A. Jyoti . 2016. “Green Synthesis of Silver Nanoparticles by Using Waste Vegetable Peel and Its Antibacterial Activities.” Journal of Pharmaceutical Sciences and Research 8, no. 5: 313.

[fsn371569-bib-0090] Singh, H. , M. F. Desimone , S. Pandya , et al. 2023. “Revisiting the Green Synthesis of Nanoparticles: Uncovering Influences of Plant Extracts as Reducing Agents for Enhanced Synthesis Efficiency and Its Biomedical Applications.” International Journal of Nanomedicine 18: 4727–4750. 37621852 10.2147/IJN.S419369 PMC10444627

[fsn371569-bib-0091] Singh, R. , M. Gupta , P. Singhal , S. Goyal , and S. K. Upadhyay . 2021. “In Vitro Antimicrobial Activities of Vegetables (Potato, Cucumber, Sweet Potato and Ginger) Peel Wastes for Ecofriendly Microbial Management.” International Journal of Botany Sciences 6: 134–137.

[fsn371569-bib-0092] Sultana, R. , and M. Misbahuddin . 2020. “Effects of Solasodine of *Solanum melongena* Peel Origin in the Treatment of Palmar Arsenical Keratosis.” Bangabandhu Sheikh Mujib Medical University Journal 13, no. 2: 47–52.

[fsn371569-bib-0093] Sultana, S. 2020. “Nutritional and Functional Properties of *Moringa oleifera* .” Metabolism Open 8: 100061. 33145489 10.1016/j.metop.2020.100061 PMC7596288

[fsn371569-bib-0094] Suvaneeth, P. , N. Nair , T. Anilkumar , et al. 2018. “Gross and Histopathologic Changes of Porcine Cholecyst Assisted Full Thickness Skin Wound Healing in Rabbits.” Journal of Livestock Science 9: 75–80.

[fsn371569-bib-0095] Talukder, P. , S. Chakraborty , M. Sarkar , A. Das , and R. Ray . 2025. “Role of Secondary Metabolites in Reducing the Negative Impact of Pest‐Induced Stress in Eggplant (*Solanum melongena* L.).” Applied Biochemistry and Biotechnology 197, no. 8: 5411–5426. 40526242 10.1007/s12010-025-05287-0

[fsn371569-bib-0096] Tasic, L. , D. Stanisic , L. G. Martins , G. C. Cruz , and R. Savu . 2022. “Insights of Green and Biosynthesis of Nanoparticles.” In Sustainable Nanotechnology for Environmental Remediation, 61–90. Elsevier.

[fsn371569-bib-0097] Thilavech, T. , and S. Adisakwattana . 2019. “Cyanidin‐3‐Rutinoside Acts as a Natural Inhibitor of Intestinal Lipid Digestion and Absorption.” BMC Complementary and Alternative Medicine 19: 1–10. 31488210 10.1186/s12906-019-2664-8 PMC6727418

[fsn371569-bib-0098] Tiwari, R. , and K. Pathak . 2023. “Local Drug Delivery Strategies Towards Wound Healing.” Pharmaceutics 15, no. 2: 634. 36839956 10.3390/pharmaceutics15020634 PMC9964694

[fsn371569-bib-0099] Tu, C. , H. Lu , T. Zhou , et al. 2022. “Promoting the Healing of Infected Diabetic Wound by an Anti‐Bacterial and Nano‐Enzyme‐Containing Hydrogel With Inflammation‐Suppressing, ROS‐Scavenging, Oxygen and Nitric Oxide‐Generating Properties.” Biomaterials 286: 121597. 35688112 10.1016/j.biomaterials.2022.121597

[fsn371569-bib-0100] Urnukhsaikhan, E. , B.‐E. Bold , A. Gunbileg , N. Sukhbaatar , and T. Mishig‐Ochir . 2021. “Antibacterial Activity and Characteristics of Silver Nanoparticles Biosynthesized From *Carduus crispus* .” Scientific Reports 11, no. 1: 21047. 34702916 10.1038/s41598-021-00520-2 PMC8548519

[fsn371569-bib-0101] Vanti, G. L. , M. Kurjogi , K. Basavesha , N. L. Teradal , S. Masaphy , and V. B. Nargund . 2020. “Synthesis and Antibacterial Activity of *Solanum torvum* Mediated Silver Nanoparticle Against Xxanthomonas Axonopodis Pv. Punicae and *Ralstonia solanacearum* .” Journal of Biotechnology 309: 20–28. 31863800 10.1016/j.jbiotec.2019.12.009

[fsn371569-bib-0102] Wypij, M. , T. Jędrzejewski , J. Trzcińska‐Wencel , M. Ostrowski , M. Rai , and P. Golińska . 2021. “Green Synthesized Silver Nanoparticles: Antibacterial and Anticancer Activities, Biocompatibility, and Analyses of Surface‐Attached Proteins.” Frontiers in Microbiology 12: 632505. 33967977 10.3389/fmicb.2021.632505 PMC8100210

[fsn371569-bib-0103] Yang, X. , E. Luo , X. Liu , B. Han , X. Yu , and X. Peng . 2016. “Delphinidin‐3‐Glucoside Suppresses Breast Carcinogenesis by Inactivating the Akt/HOTAIR Signaling Pathway.” BMC Cancer 16: 1–8. 10.1186/s12885-016-2465-0 PMC4937537 27388461

[fsn371569-bib-0104] Yazarlu, O. , M. Iranshahi , H. R. K. Kashani , et al. 2021. “Perspective on the Application of Medicinal Plants and Natural Products in Wound Healing: A Mechanistic Review.” Pharmacological Research 174: 105841. 34419563 10.1016/j.phrs.2021.105841

[fsn371569-bib-0105] Yu, Y. , Z. Zhang , and C. Chang . 2022. “Chlorogenic Acid Intake Guidance: Sources, Health Benefits, and Safety.” Asia Pacific Journal of Clinical Nutrition 31, no. 4: 602–610. 36576278 10.6133/apjcn.202212_31(4).0003

[fsn371569-bib-0106] Yusuf, A. , A. R. Z. Almotairy , H. Henidi , O. Y. Alshehri , and M. S. Aldughaim . 2023. “Nanoparticles as Drug Delivery Systems: A Review of the Implication of Nanoparticles' Physicochemical Properties on Responses in Biological Systems.” Polymers 15, no. 7: 1596. 37050210 10.3390/polym15071596 PMC10096782

[fsn371569-bib-0107] Zafar, M. G. , M. Abbas , W. Haider , et al. 2025. “Molecular Docking, Dynamics, and Pharmacokinetics of *Curcuma longa* Phytochemicals: A Promising Avenue for Parkinson's Therapy.” Journal of the Indian Chemical Society 102: 101790.

[fsn371569-bib-0108] Zhou, P. , P. K. Theil , D. Wu , and K. E. B. Knudsen . 2018. “In Vitro Digestion Methods to Characterize the Physicochemical Properties of Diets Varying in Dietary Fibre Source and Content.” Animal Feed Science and Technology 235: 87–96.

[fsn371569-bib-0109] Zia‐ur‐Rehman, Z. , M. Islam , and W. Shah . 2003. “Effect of Microwave and Conventional Cooking on Insoluble Dietary Fibre Components of Vegetables.” Food Chemistry 80, no. 2: 237–240.

